# 5′ leader defects drive persistent HIV-1 viremia on long-term ART

**DOI:** 10.1038/s41467-026-73475-5

**Published:** 2026-06-08

**Authors:** Julia R. Box, Angelica Camilo-Contreras, Filippo Dragoni, Feng Yun Yue, Vitaliy Matveev, Jackson Foley, Jianwei Zhang, Yan Wei Mok, Marlene DeSousa, Jun Lai, Zachary Mulcare, Zachary Bakewell, Sebastien Poulin, Frederic Chano, Claude Fortin, Cecile Tremblay, Joel N. Blankson, Sonya Krishnan, Ethel D. Weld, Christie Basseth, Matthew M. Hamill, Christopher J. Hoffmann, Eileen P. Scully, Joyce L. Jones, Andrea L. Cox, Wissam El Atrouni, Beverly Sha, Janet D. Siliciano, Robert F. Siliciano, Robert Reinhard, Jesper D. Gunst, Mario Ostrowski, Frank Maldarelli, Colin Kovacs, Francesco R. Simonetti

**Affiliations:** 1 Department of Medicine, Johns Hopkins University School of Medicine, Baltimore, MD USA; 2 Department of Medicine, University of Toronto, St. Michael’s Hospital, Unity Health, Toronto, Ontario, Canada; 3 Maple Leaf Medical Clinic, Toronto, Ontario, Canada; 4 Clinique L’Agora, Montréal, Québec, Canada; 5 Centre de Recherche du Centre Hospitalier de l’Université de Montréal, Montréal, Québec, Canada; 6 Département de Microbiologie, Immunologie et Infectiologie, Université de Montréal, Montréal, Québec, Canada; 7 Division of Infectious Diseases, The University of Kansas Medical Center, Kansas City, KS USA; 8 Rush University Medical Center, Division of Infectious Diseases, Chicago, IL USA; 9 Howard Hughes Medical Institute, Baltimore, MD USA; 10 San Francisco, CA USA; 11 Aarhus University, Department of Clinical Medicine, Aarhus, Denmark; 12 Aarhus University Hospital, Department of Infectious Diseases, Aarhus, Denmark; 13 National Cancer Institute, Frederick, MD USA

**Keywords:** Retrovirus, Infection, HIV infections

## Abstract

Traces of HIV-1 RNA can persist in plasma despite long-term suppressive antiretroviral therapy (ART). Some individuals develop nonsuppressible viremia (NSV), characterized by detectable HIV-1 RNA that raises concerns for virological failure, pathogenesis, and transmission. The sources of NSV remain poorly defined, in part due to limited tools to characterize plasma HIV-1 RNA. Both infectious and defective proviruses, including those with defects in the 5′ Leader (5′L), can contribute to NSV, but their relative contributions have not been quantified. Here we show that in over 50 participants, plasma viremia is markedly driven by highly clonal HIV-1 RNA populations carrying defects in the 5′L. Across individuals, dominant clones with 5′L defects clustered around the major splice donor (MSD) accounted for the vast majority of circulating HIV-1 RNA. To enable rapid, scalable profiling, we developed CLAWS (Capturing 5′ Leader Anomalies Without Sequencing), a digital PCR assay that distinguishes intact from defective 5′L RNA. CLAWS recapitulated sequencing-based estimates and detected low-abundance defective RNA early after ART initiation, revealing that defective genomes emerge early and become predominant during long-term therapy. These findings identify 5′L-defective genomes as the predominant driver of NSV and establish CLAWS as a practical tool for monitoring viremia in clinical and cure-related settings.

## Abstract (web summary)

Traces of HIV-1 RNA remain in the plasma despite years of antiretroviral therapy (ART), sometimes causing nonsupressible viremia (NSV). Here, the authors show that 5′Leader-defective RNA is the predominant driver for NSV in ART-treated individuals.

## Introduction

Despite years of effective antiretroviral therapy (ART), HIV-1 persists due to a reservoir of infected CD4⁺ T cells harboring replication-competent proviruses that can reignite viremia if ART is interrupted^1,2^. The reservoir is exceptionally stable^3^, with minimal decay even after two decades of suppressive therapy^4^, owing to low proviral inducibility^5^ and proliferation of infected cells in response to antigenic or homeostatic stimuli^6–8^.

HIV-1 persistence is further complicated by the high abundance of defective proviruses, which constitute over 90% of integrated genomes in people with HIV (PWH)^9,10^. Once thought inert, defective proviruses can be transcriptionally and translationally active^11^, producing RNA, proteins^12–14^, and viral particles^15^. Because of their abundance and residual transcriptional activity, defective proviruses can (i) affect the interpretation of PCR-based assays that quantify HIV-1^16,17^, (ii) contribute to residual inflammation and immune activation despite effective therapy^18,19^, and (iii) produce irrelevant ‘decoy’ epitopes that distract and exhaust the immune system^11^, which may thwart the induction of HIV-1 control by novel therapeutic interventions. Thus, even if they cannot lead to viral rebound, defective proviruses are still clinically relevant.

Trace levels of plasma HIV-1 RNA, also referred to as residual viremia (RV), are detected in more than half of PWH on ART^20–22^. While typically low (1–3 copies/mL), some individuals exhibit persistently detectable levels ( > 20 copies/mL), referred to as nonsuppressible viremia (NSV)^15,23–25^. At present, there is no formally standardized definition for NSV. Rather, the term has been used to refer to low-level viremia despite ART adherence and absence of drug resistance, with variability across studies in viral load thresholds and duration criteria^26^. RV and NSV are reflections of the same phenomenon, the release of viral particles from infected cells that persist because of clonal expansion^15,23,24,27,28^. NSV, raises concerns for virologic failure, transmission, and immune activation, complicates ART management, causing anxiety and ultimately affecting the quality of life of PWH^26,29^. It also increases healthcare utilization and can strain the PWH-provider relationship. Its prevalence appears to be increasing, likely reflecting improved detection due to more sensitive clinical assays and the growing population on long-term ART^30^. NSV also complicates the design and inclusion criteria for HIV-1 cure studies, potentially excluding from clinical trials otherwise eligible PWH^29^.

We and others have shown that proviruses underlying NSV can have defects in the 5’Leader (5’L) region^15,24,31^. These defects are introduced during minus-strand DNA synthesis by reverse transcriptase and are favored by microhomology repeats, homopolymeric tracts, and RNA secondary structures within the 5′L^32^. The 5’L orchestrates essential steps in the viral life cycle, including reverse transcription, HIV-1 RNA transcription, splicing, dimerization, and packaging^33–36^. Thus, even minor disruptions in this highly structured RNA region can curb replication competence. We previously characterized four cases of NSV^15^ caused by proviruses with small deletions or point mutations affecting the major splicing donor (MSD, also known as D1), from which virtually all spliced transcript isoforms originate^37,38^. When the MSD is mutated or missing, alternative/cryptic donors take over but fail to preserve the normal splicing landscape, producing non-infectious viral particles with reduced Envelope glycoprotein incorporation^11,15^.

Previous studies were limited by small sample sizes, leaving the prevalence and impact of 5′L defects uncertain. To address this gap, we analyzed plasma from a larger cohort of PWH with NSV and found that 5′L-defective proviruses dominate persistent HIV-1 RNA. Building on this insight, we developed CLAWS, a digital PCR (dPCR) assay that distinguishes intact from defective 5′L RNA without sequencing, providing a rapid, cost-effective tool for investigating NSV and HIV-1 cure-related research.

## Results

### HIV-1 RNA variants contributing to NSV are highly clonal

**Fig. 1:**
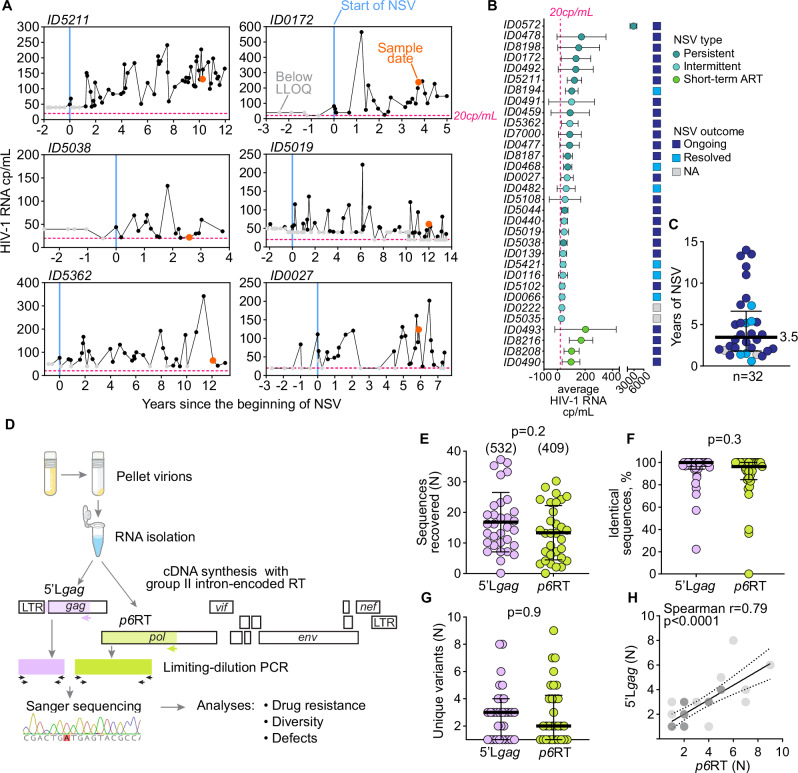
NSV is driven by few predominant plasma clones. **A** Longitudinal HIV-1 RNA measurements of six representative participants with persistent or intermittent NSV; additional participant data is shown in Supplementary Fig. S1; values below the limit of quantification are indicated in gray, the blue bar indicates the start of NSV, and the orange circle indicates the sample collection date; the red dotted line indicates the limit of quantification of 20 copies/mL, typical of the most sensitive clinical assays. **B** Average of HIV-1 RNA in plasma measured from the start of NSV until resolution or the end of follow-up for the 32 study participants; symbols indicate average values and error bars indicate standard deviation. **C** Duration of NSV observation (*n* = 32); symbols are color-coded based on whether NSV was ongoing or resolved by the end of follow-up; error bars indicate median and interquartile range. **D** Experimental design to obtain single genome sequences from HIV-1 RNA in plasma. **E** Number of sequences recovered from each participant (*n* = 31); total sequences recovered are indicated in parentheses; horizontal bars indicate mean and standard deviation; p value obtained by two-sided parametric* t* test. **F** Percentage of identical sequences in the plasma from each participant (*n* = 31); horizontal bars indicate median and interquartile range; *p* value obtained by two-sided Mann-Whitney test. **G** Number of viral variants in plasma for each participant (*n* = 31); horizontal bars indicate median and interquartile range; p value obtained by two-sided Mann-Whitney test. **H** Two-sided Spearman correlation analysis of the number of variants estimated based on 5’L*gag* versus *p6*RT (*p* < 0.0001); dotted lines indicate 95% confidence intervals.

We analyzed plasma samples from 32 PWH on ART with NSV, defined as persistent or intermittent detectable plasma HIV-1 RNA for at least six months despite no evidence of poor adherence, drug resistance by HIV DNA genotype, or drug–drug interactions that might indicate incomplete suppression of viral replication. In some participants, attempts of optimization or intensification of ART failed to reduce viremia, as previously described^15,39^. The cohort had a median age of 61 years (interquartile range, 56–68) and consisted primarily of male (29/32) and Caucasian (19/32) participants. Additional demographic and clinical characteristics are summarized in Supplementary Table S1. For most individuals, NSV emerged after years of previously undetectable HIV-1 RNA and persisted either continuously (always above the detection limit) or intermittently over prolonged intervals (Fig. 1A, B and Supplementary Fig. S1). In contrast, four participants never achieved undetectable viremia after ART initiation, despite an initial multi-log decline in HIV-1 RNA and CD4^+^ T cell recovery (Supplementary Fig. S1B). The median duration of observed NSV was 3.5 years (range, 0.6–13.4), and in six participants, plasma viral load eventually returned to undetectable levels during follow-up (Fig. 1C and Supplementary Fig. S1).

To characterize viral sequences contributing to NSV, we pelleted plasma by centrifugation, isolated RNA, synthesized cDNA with HIV-1–specific primers, and performed limiting-dilution nested PCR spanning (i) the 5′L and *gag* and (ii) the p6, protease, and reverse transcriptase (RT) regions (Fig. 1D). To improve efficiency, we used a group II intron–encoded reverse transcriptase (InduroRT, NEB) for cDNA synthesis and VeriFi polymerase for PCR. Sanger sequencing data were quality-controlled and analyzed for clonality, 5′L defects, and drug-resistance mutations in protease or RT. We recovered 532 5′L*gag* and 409 *p6*RT sequences from 31 of 32 participants (median, 16 and 13 sequences per participant, respectively; Fig. 1E). Plasma HIV-1 RNA sequences were highly clonal, with a median of 100% (IQR 94–100%) and 96% (IQR 84–100%) identical sequences for 5′L*gag* and *p6*RT (Fig. 1F), corresponding to 91 and 87 unique variants, respectively. In agreement with previous studies^15,23,24,27^, virus in plasma derived from only a few predominant variants, with a median of 3 and 2 variants for 5′L*gag* and *p6*RT, respectively (Fig. 1G and Supplementary Fig. S2A), showing robust correlation between the two regions analyzed (Fig. 1H). Mutations associated with drug resistance to protease or RT inhibitors were rare, likely archived during previous virological failures, and did not impact current ART (Supplementary Table S1 and Supplementary Fig. S2B), consistent with previous studies on RV^40^ and viral blips^41^.

Together, these results confirm that NSV is driven by HIV-1 gene expression and virion release from clonally expanded infected cells, rather than ongoing replication.

### 5′ Leader–defective HIV-1 RNA is the predominant driver of NSV

**Fig. 2:**
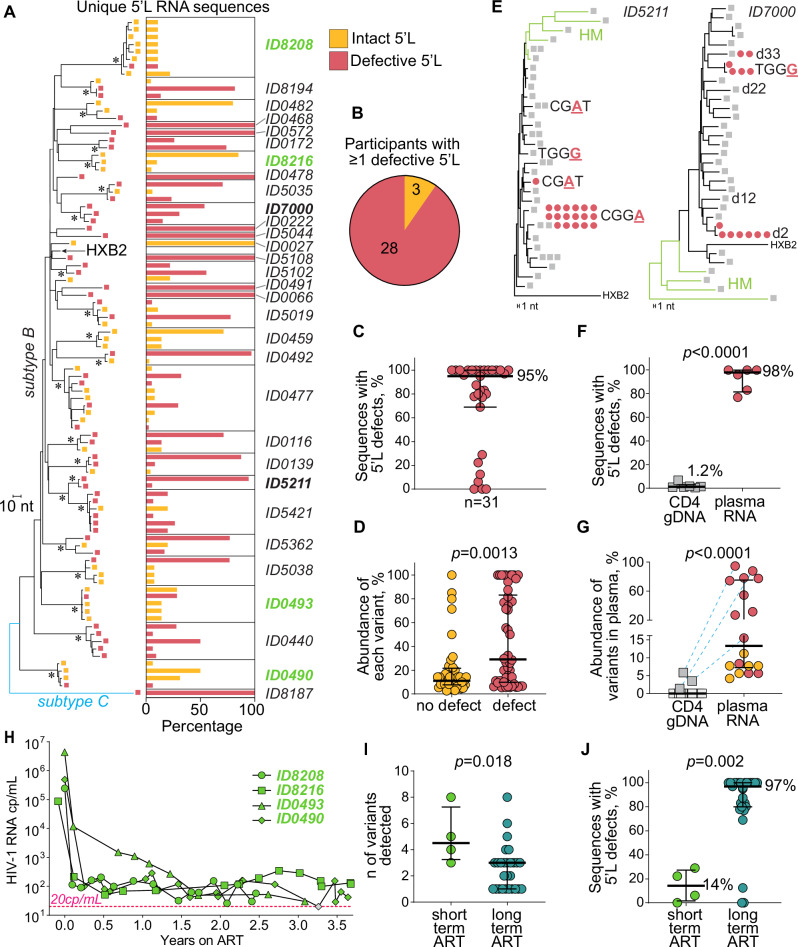
5′ Leader–defective sequences dominate HIV-1 RNA in plasma in PWH on long-term ART. **A** Maximum likelihood phylogenetic tree of 5’L*gag* unique sequences from 31 participants, using a GTR + G + I substitution model; the tree is rooted to one subtype C variant from participant ID8187; nodes with bootstrapping values above 85 are indicated with a star symbol; 5’L intact and defective sequences are indicated in orange and red, respectively; bar charts indicate the percentage of each variant. **B** Pie chart showing the number of participants in whom we found at least one 5’L-defective sequence. **C** Percentage of 5’L-defective sequences found in each participant; horizontal bars indicate median and interquartile range. **D** Percentage abundance of each variant (*n* = 91); horizontal bars indicate median and interquartile range; p value obtained with two-sided Mann-Whitney test (*p* = 0.0013). **E** Maximum likelihood phylogenetic tree of 5’L*gag* sequences from plasma RNA (circles) and CD4^+^ T cell proviral DNA (gray squares); MSD defects are shown; hypermutated sequences are indicated with green tree branches. **F** Percentage of 5’L-defective sequences in plasma versus proviruses from six participants; horizontal bars indicate median and interquartile range; p value obtained by paired two-sided *t* test (*p* < 0.0001). **G** Percentage abundance of variants found in plasma from six participants and their corresponding abundance in CD4^+^ T cells; horizontal bars indicate median and interquartile range; p value obtained with two-sided Mann-Whitney test (*p* < 0.0001). **H** Plasma HIV-1 RNA measurements of four participants who did not achieve undetectable viremia despite > 3 years of ART. **I**,** J** Comparison of the number of variants detected (**I**) and percentage of 5’L defects (**J**) between participants with short (*n* = 4) versus long-term ART (*n* = 27); horizontal bars indicate median and interquartile range; *p*-value obtained with two-sided Mann-Whitney test (*p* = 0.018 and *p* = 0.002, respectively).

To quantify the contribution of 5′L defects to plasma HIV-1 RNA, we calculated the proportion of unique variants among all recovered sequences. Unique 5′L variants from each participant were phylogenetically related and accounted for between 3% and 100% of sequences (Fig. 2A). Strikingly, 5′L-defective sequences—defined by mutations within the MSD ([T/C]GGTGAG) or deletions outside regions of length variation—were identified in 28 of 31 participants (90%, Fig. 2B). In most individuals, these defective variants dominated viremia, with a median abundance of 95% (IQR 69–100%, Fig. 2C and Supplementary Fig. S3). In 9 participants, NSV was caused by a single 5′L-defective plasma clone; only seven participants had a low proportion ( < 30%) of defective variants (Supplementary Fig. S3). Across all individuals, 5′L-defective sequences were significantly more abundant than sequences without 5’L defects (*p* = 0.0013, Fig. 2D).

To assess whether these defective virions were overrepresented in plasma because of highly expanded infected cell clones, we analyzed 249 5’L*gag* proviral sequences from peripheral blood CD4^+^ T cells in six individuals (on average, 41 sequences per participant, Supplementary Fig. S4A). Proviral populations were more diverse and contained fewer identical sequences (Fig. 2E and Supplementary Fig. S4B, C), consistent with prior reports^15^. As expected, APOBEC3G/F hypermutated genomes were detected only in proviral DNA (Supplementary Fig. S4D), reflecting the need for intact open reading frames ORFs, especially Gag^42,43^, to produce viral particles. Although 5′L-defective proviruses were detectable, they were rare (1.2%) compared to plasma RNA (98%, *p* < 0.0001, Fig. 2F). Matching DNA sequences for plasma 5′L clones were found only in three instances (Fig. 2G), indicating that NSV-producing proviruses are rare among circulating infected cells despite clonal expansion.

A subset of participants (*n* = 4) showed a pattern in which plasma HIV-1 RNA declined after ART initiation but never reached undetectable levels (Fig. 2H and Supplementary Fig. S1B). Their plasma sequences were significantly more diverse (*p* = 0.018; Fig. 2I) and carried fewer 5′L defects (14%, *p* = 0.02; Fig. 2J) than those from individuals who developed NSV after years of suppression (97%). These differences suggest that NSV emerging shortly after ART initiation may be driven by different mechanisms, such as a larger reservoir size, and support the hypothesis that 5’L defective plasma clones become enriched over time as the proviral landscape undergoes selection^4,44^.

### Recurring deletions and mutations cluster within the MSD

**Fig. 3:**
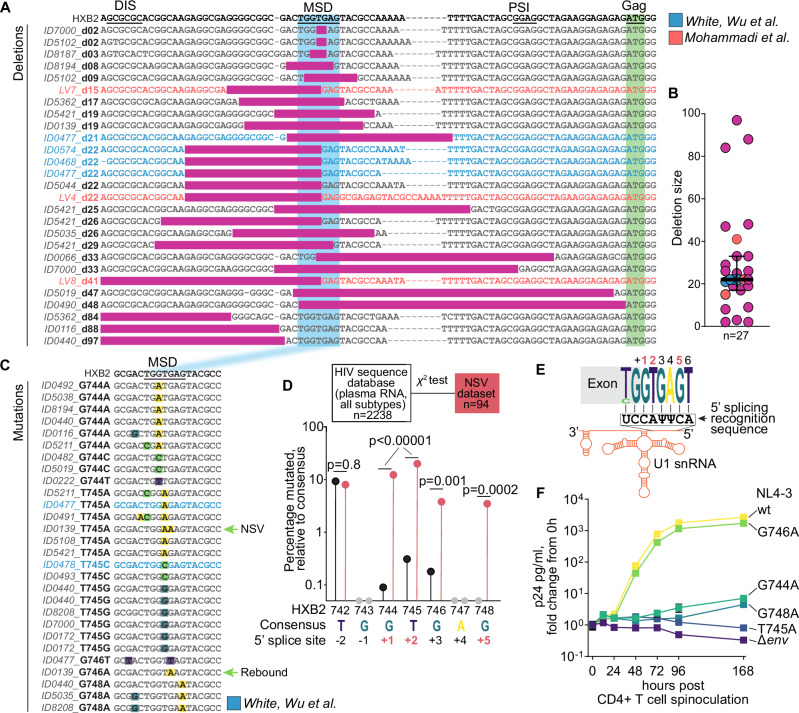
Deletions and single nucleotide mutations are narrowly focused on the MSD, affecting HIV-1 RNA splicing. **A** Detail of variants with deletions; data previously published are highlighted in red and blue; DIS, dimerization signal; PSI, packaging signal. **B** Distribution of deletion size, indicating missing nucleotides; horizontal bars indicate median and interquartile range. **C** Detail of variants with single nucleotide mutations found in the MSD; sequences from ID0139 are highlighted in green, to show that the G746A mutation was present in both NSV and rebound variants. **D** Percentage of sequences with mutations at each MSD position; black lollipops indicate a control dataset from the HIV Sequence Database, while those in red indicate the NSV dataset; difference in proportion was assessed by two-sided Chi Square test (*p*-values indicated in the figure); the most conserved 5’ splice site positions across thousands of human genes are indicated in red (adapted from Roca et al.^48^). **E** Graphic representation of the interaction between the HIV-1 5’ exon-intron junction and the U1 small nuclear RNA. **F** Replication of NL4-3 viruses in which we introduced mutations at MSD positions of interest, measured a fold increase in supernatant p24 from 0 h post spinoculation; wildtype (wt) and Δ*env* viruses were used as positive and negative controls, respectively; data from three technical replicates.

Among the 91 unique 5′L RNA variants recovered, 26% harbored deletions and 30% contained single-nucleotide mutations within the MSD site (Fig. 3). The sequenced *gag* coding region (HXB2 amino acids 1–334) was intact in all but one variant, which carried multiple APOBEC3G-mediated G-to-A mutations. This hypermutated species, found in participant ID0116 and recovered from multiple plasma samples (*n* = 5), was not the result of genomic DNA contamination, as confirmed by control reactions lacking reverse transcriptase. Given the stop codons in Gag, we consider packaging of this RNA into virions unlikely; instead, its release into plasma likely occurs via extracellular vesicles or cytolysis of infected cells, as previously described^45^.

Analysis of 20 newly identified deletions, together with 7 previously reported (Fig. 3A), revealed a predominant involvement of the MSD. Although deletion size varied from 2–97 nucleotides (mean 22; Fig. 3B), 88% (24/27) disrupted the MSD and only rarely extended into adjacent dimerization (DIS) or encapsidation (PSI/Ψ) stem loops^36^. All deletions preserved the *gag* gene start codon. Notably, the 22-nucleotide deletion (Δ22) has now emerged as a dominant plasma clone in five individuals with NSV^15,24^.

We also identified 27 variants with recurrent point mutations at specific MSD nucleotides (Fig. 3C), most frequently at HXB2 positions G744 (*n* = 9) and T745 (*n* = 14) — the first two intronic nucleotides following the exon–intron junction. These residues are highly conserved in human genes because they form the canonical GT dinucleotide essential for U1 snRNA recognition of the 5′ splice site^46–48^ (5′ss). Mutations at T745 have been shown to dramatically decrease viral fitness and have been reported in viruses driving persistent viremia^15,31^. Additional substitutions occurred at G746 (*n* = 2) and G748 (*n* = 3). To assess their prevalence, we screened 2238 publicly available plasma RNA sequences. While a known polymorphic change at T742C was equally represented ( ~ 10%, *p* = 0.8), substitutions at positions 744, 745, 746, and 748 were significantly enriched in NSV-associated sequences (Fig. 3D), suggesting a functional impact on 5′ss recognition. Positions + 3 and + 4 (relative to the exon-intron junction) interact with pseudo-uridines of the U1 snRNA, conserved from yeast to humans, binding to both A or G bases^49^ (Fig. 3E). Consistent with this, all sequences from participant ID0139 carried the G746A mutation (Fig. 3C) — a rare but apparently tolerated change.

To directly test the impact of these mutations on viral fitness, we infected activated CD4^+^ T cells with a panel of NL4-3 mutants (G744A, T745A, G746A, G748A) alongside wild-type (WT) and envelope-defective (Δ*env*) controls (Fig. 3F). Only WT and G746A viruses resulted in an exponential increase in p24, confirming that the G746A mutation does not affect viral replication. In contrast, T745A behaved like Δ*env*, producing negligible change in p24, and G744A and G748A showed p24 concentrations only ~ 5-fold above baseline after 7 days — consistent with near-complete loss of replicative capacity.

Taken together, these findings show that plasma viruses from individuals with NSV are enriched for deletions and point mutations that disrupt HIV-1 RNA splicing at the MSD. Although these alterations compromise replication, they may enable transcriptionally active but replication-defective proviruses to evade clearance and persist, contributing disproportionately to viremia during ART.

### A single digital PCR assay can identify 5’L defects

**Fig. 4:**
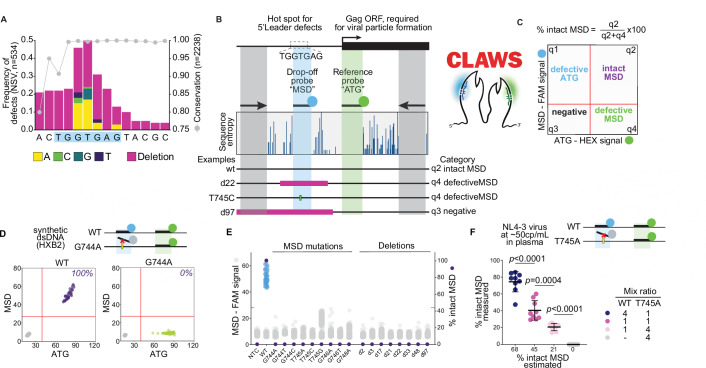
A single digital PCR assay allows to distinguish intact and defective 5’L RNA. **A** Frequency of defects detected in HIV-1 RNA from participants with NSV paired with the analysis of sequence conservation across circulating HIV-1 strains; shaded area indicates the MSD sequence; the analysis is based on 534 RNA sequences from NSV and 2238 RNA sequences from the Los Alamos National Laboratory database. **B** Assay design of CLAWS (Capturing 5’ Leader Anomalies Without Sequencing); the bar chart below indicates sequence entropy; representative intact and defective 5’L leaders are indicated at the bottom. **C** Schema of a 2-dimention (2D) digital PCR plot showing how partitions are scored based on fluorescence of the MSD and ATG probes; q indicates the quadrants defined by the thresholds (red lines). **D** Example of CLAWS read out and the impact of a single nucleotide mutation using synthetic double-stranded DNA (dsDNA); the number at the top right corner indicates the percentage of HIV-1 positive partitions with intact MSD; panels represent a single replicate each. **E** Single point mutations and deletions cause complete loss of MSD probe signal, correctly parsing intact and defective RNA (also see Supplementary Fig. S4A). **F** NL4-3 virus stocks generated in vitro were diluted into plasma from pooled healthy donors; wildtype (WT) and T745A NL4-3 variants were mixed at different ratios at tested by CLAWS; each symbol represents a replicate aliquot of plasma; data obtained from 8-9 aliquots of plasma per each ratio; horizontal bars indicate mean and standard deviation; *p*-value obtained by parametric two-sided *t* test (*p*-values indicated in the figure).

Recovering single-genome sequences from low-copy plasma virus is costly and labor-intensive. To address this, we designed a single dPCR assay capable of both quantifying HIV-1 RNA and determining 5′L intactness. Sequence analysis revealed that 5′L defects in plasma cluster tightly around the major splice donor (MSD), a region otherwise highly conserved and positioned immediately upstream of the *gag* open reading frame (Fig. 4A). This sequence architecture suggested a streamlined “drop-off” digital PCR design: one fluorescent probe targeting a highly conserved *gag* start site (“ATG”, reference probe) and a second probe binding the MSD only when intact (“MSD”, drop-off probe) (Fig. 4B). This strategy, often used in liquid biopsy assays to detect cancer mutations in cell-free DNA^50,51^, leverages the fact that Gag is required for virion assembly and RNA packaging, and is therefore preserved in most plasma genomes^43^. The MSD probe was engineered for maximal specificity — 10 nucleotides in length, incorporating locked nucleic acids (LNA), modified bases containing a methylene bridge bond between the 2′ oxygen and the 4′ carbon of the pentose ring to increase melting temperature^52^ and improve discrimination against common mutations (Supplementary Table S2).

The workflow mirrors that for single-genome sequencing: RNA is isolated from one or multiple 1 mL aliquots of plasma, reverse transcribed, and analyzed by dPCR. Poisson-based quantification yields total *gag* RNA copies, while the multiplex readout calculates the fraction of intact 5′L RNA (quadrant 2) relative to total *gag* RNA (quadrants 2 + 4) (Fig. 4C).

We validated the assay, which we named CLAWS (Capturing 5′ Leader Anomalies Without Sequencing), using a panel of synthetic double-stranded DNA templates of the HXB2 reference containing 9 single-nucleotide mutations and 8 deletions recovered from individuals with NSV. CLAWS correctly distinguished intact from defective sequences at single-nucleotide resolution, providing accurate intactness estimates for all synthetic templates (Figs. 4D, E, and Supplementary Fig. S5A). Testing with NL4-3 virus stocks carrying the T745A mutation at defined ratios confirmed CLAWS can resolve mixtures of intact and defective 5′L RNA (Fig. 4F). Some large deletions spanning primer-binding sites (e.g., Δ97) could not be amplified and appeared as double-negative partitions (quadrant 3, Supplementary Fig. S5A).

Assay sensitivity was optimized by performing cDNA synthesis separately with InduroRT and the CLAWS reverse primer, which significantly increased HIV-1 RNA recovery compared to one-step dPCR (Supplementary Fig. S5B, C). Using this protocol, CLAWS showed excellent linearity (Spearman *r* = 1,* p* = 0.0028) in serial dilutions of wild-type NL4-3 spiked into pooled plasma from HIV-1 negative donors, with a 95% limit of detection of 9 copies/mL (Supplementary Fig. S5D). HIV-1 RNA standards from the Virology Quality Assurance (VQA) program confirmed reproducibility: all ten 20 copies/mL controls were detected (mean 14.6 copies/mL, 95% CI 10.4–19.5) and all four 0 copies/mL controls were negative (Supplementary Fig. S5E).

These validation experiments demonstrate that CLAWS is highly specific, sensitive, and able to distinguish intact from defective 5′L RNA at single-nucleotide resolution, providing a rapid and scalable alternative to sequencing for quantifying low-level plasma HIV-1 RNA and parsing its 5’L integrity.

### CLAWS strongly correlates with 5′Lgag RNA sequencing

**Fig. 5:**
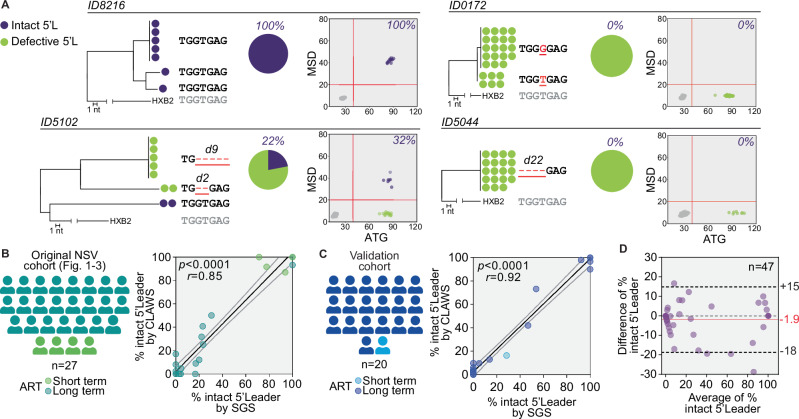
CLAWS strongly correlates with 5’L RNA sequencing data from clinical samples. **A** Paired 5’L*gag* and CLAWS data of four representative participants with NSV; panels on the left show neighbor-joining trees; MSD sequence is indicated in text to tree taxa; pie charts indicate the percentage of intact 5’L sequences; panels on the right show CLAWS 2D plots. **B** Schematic representation of the original NSV cohort used to test CLAWS; the left panel shows a two-sided Spearman correlation analysis of 5’Leader intact RNA measured by single genome sequencing and CLAWS (*p* < 0.0001); gray lines indicate 95% confidence interval. **C** Schematic representation of the validation cohort used to confirm CLAWS performance; see Supplementary Fig. S7 and Supplementary Table S2 for additional characterization; two-sided Spearman correlation is indicated in the right panel (*p* < 0.0001). **D** Bland-Altman plot of combined data from (**B** and **C**), indicating the mean bias between the two methods (red line); black dashed lines indicate 95% confidence intervals of the mean bias.

Following assay validation on HIV-1 laboratory standards, CLAWS was applied to clinical samples. Plasma from ART-naïve individuals contained 100% intact 5′L RNA, consistent with active replication (Supplementary Fig. S5F). In samples from PWH with NSV, intact RNA proportions varied but closely matched 5’L*gag* sequencing results (Fig. 5A). Across 27 paired samples, the two methods were highly correlated (Spearman *r* = 0.84, *p* < 0.0001; Fig. 5B) and yielded comparable estimates of intact 5’L RNA (4.5% vs. 8.0%, *p* = 0.28; Supplementary Fig. S6).

To evaluate the generalizability of CLAWS beyond the initial cohort, we assessed its performance in an independent validation cohort of 20 individuals with NSV who had not been included in prior 5′L analyses (Fig. 5C and Supplementary Fig. S6). Participants were referred from multiple HIV clinics based solely on persistent or intermittent detectable plasma HIV-1 RNA in the preceding months and were not pre-characterized for 5′L defects or other virologic or clinical features (Supplementary Fig. S6A, B and Supplementary Table S2). As in the original cohort, we quantified the proportion of 5′L–intact plasma HIV-1 RNA using both 5′Lgag single-genome sequencing (*n* = 482 new sequences) and CLAWS. The median percentage of 5′L–intact RNA was 2.5% (IQR, 0–82%) by single-genome sequencing and 9.7% (IQR, 0.8–85.8%) by CLAWS (*p* = 0.4, Supplementary Fig. S6C), comparable to estimates obtained in the initial cohort. The two assays again demonstrated strong concordance (Spearman *r* = 0.9, *p* < 0.0001; Fig. 5C). In only two instances, we identified HIV-1 variants with large deletions or point mutations predicted to impair detection by CLAWS (Supplementary Fig. S6D). Overall, systematic bias between sequencing and CLAWS was minimal (mean difference − 1.9%, 95% CI − 18% to + 15%; Fig. 5D). Notably, we identified a sixth individual whose NSV was caused by a predominant clone harboring the recurring d22 deletion (VC1005, Supplementary Fig. S6D). Together, these findings demonstrate that CLAWS reliably distinguishes 5′ Leader–intact from defective plasma HIV-1 RNA in an independent cohort and support its applicability as a robust alternative to single genome sequencing.

### CLAWS reveals progressive enrichment of 5’L defective RNA in plasma over time on ART

**Fig. 6:**
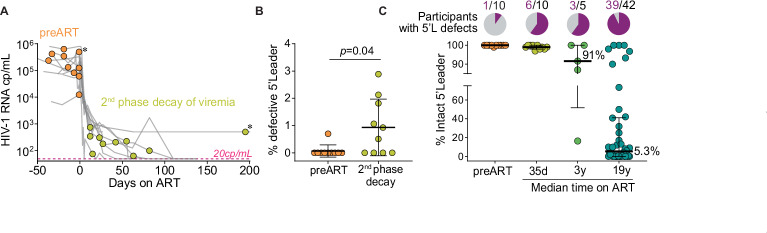
CLAWS reveals progressive increase of 5’L-defective RNA in plasma over time on ART. **A** Summary of HIV-1 RNA in plasma from 10 individuals starting ART (see individual plots in Supplementary Fig. S7A); orange and yellow symbols indicate samples collected before ART and during the second phase decay of viremia, respectively; star symbols indicate values outside of the limit of quantification. **B** Percentage of 5’L-defective RNA measured by CLAWS in each participant (*n* = 10); horizontal bars indicate mean and standard deviation; *p*-value obtained by non-parametric two-sided Wilcoxon test (*p* = 0.04). **C** Percentage of intact 5’L RNA in plasma from PWH grouped by time on ART; pie charts at the top indicate the fraction of participants from whom we detected 5’L-defective RNA; horizontal bars indicate median and interquartile range.

Although defective proviruses carrying 5′L defects are transcriptionally active, their contribution to viremia throughout HIV-1 infection remains unknown. We hypothesize that these variants accumulate during long-term treatment and become disproportionately represented in individuals with NSV. To test this hypothesis, we investigated whether CLAWS could detect 5′L defects before ART and during early viral decay, when plasma HIV-1 RNA drops to ~ 1% of the pre-ART set point^53^. Pairs of longitudinal plasma samples from 10 participants who were highly monitored from ART initiation to sustained suppression (Fig. 6A and Supplementary Fig. S7A). Plasma viral load estimates by CLAWS strongly matched clinical measurements (Spearman* r* = 0.97, *p* < 0.0001; Supplementary Fig. S7B). Pre-ART, 5′L defects were rare (1 of 10 participants; 0.7% defective MSD). During second-phase decay, defects appeared in 6 of 10 participants with a significant increase in abundance (mean, 0.93% ± 1 SD; Fig. 6B and Supplementary Fig. S7C, D), suggesting that clearance of short-lived, productively infected cells unmasks low-level contribution from defective proviruses within the first month of ART. In the combined NSV cohorts (*n* = 47), the proportion of 5′ Leader–intact RNA was significantly lower in individuals on long-term ART (median 19 years) compared with those on short-term ART (median 3 years), decreasing from 91.7% to 5.3% (*p* = 0.006; Fig. 6C). These findings further support the progressive enrichment of 5′ Leader–defective variants in plasma over time, consistent with the slower decay kinetics of defective proviruses during suppressive therapy. Together, these results show that CLAWS not only mirrors sequencing accuracy but can also capture the early emergence and long-term enrichment of defective RNA, making it a scalable tool for studying HIV-1 RNA dynamics and NSV.

Although CLAWS was designed for plasma HIV-1 RNA, it can also quantify 5’L-intact (MSD^+^ATG^+^) and defective (MSD^-^ATG^+^) HIV-1 DNA from CD4^+^ T cells (Supplementary Fig. S8). In a subset of 10 participants on long-term ART (median 19 years on ART, range 13-24), CLAWS revealed that 95% of ATG^+^ proviruses retained an intact MSD—a striking contrast to plasma HIV-1 RNA, where only 8.4% were MSD-intact (*p *= 0.004, Supplementary Fig. S8A), in agreement with the 5’L*gag* sequence data (Fig. 2F). To relate these findings to the broader proviral landscape, we compared the frequency of 5’L-defective HIV-1 DNA copies measured by CLAWS with the frequency of intact and total proviruses quantified by the Intact Proviral DNA Assay^17^ (IPDA). The two methods have partially overlapping primers and probes in the 5’L region (Supplementary Fig. S8B). In addition, proviruses are not equally scored by the two assays, given that CLAWS only targets the 5’L and the first portion of *gag*. For example, proviruses scored as intact or 3’ deleted by IPDA would be both classified as 5’L intact by CLAWS (Supplementary Fig. S8C). The median frequency of 5’L-defective proviruses was 100 copies/10^6^ CD4^+^ T cells (IQR, 12-178), comparable to that of intact proviruses (214, IQR 152-300, *p* = 0.13) but significantly lower than that of 5’L-intact DNA (1079, *p* = 0.0027, Supplementary Fig. S8D), which largely represents defective genomes^16,17^. Consistent with previous near-full-length sequencing studies^9,10,54^, the percentage of 5’L-defects relative to total HIV-1 DNA was low (2.5%, IQR 0.8-11) and not significantly different than intact proviruses (9.6%, IQR 4-18, *p* = 0.16, Supplementary Fig. S8E). Together, these findings confirm that 5′L-defective proviruses are rare within the proviral reservoir, suggesting that their disproportionate contribution to plasma HIV-1 RNA in NSV cannot be explained solely by higher clonal expansion. Instead, they point to distinct biological properties that favor virus production from 5′L-defective genomes relative to those fully intact.

### Application of CLAWS to a participant in an HIV-1 cure clinical trial

Determining the source of plasma virus is essential for interpreting outcomes of HIV-1 cure-related trials involving analytical treatment interruption (ATI). We applied sequencing and CLAWS to investigate NSV and viral rebound in a participant from the TITAN trial—a randomized, placebo-controlled, double-blind phase 2a study testing the TLR9 agonist lefitolimod and two broadly neutralizing antibodies (bNAbs), 3BNC117 and 10-1074, for virologic control during ATI^55^. The trial found no added benefit of lefitolimod, whereas bNAbs delayed rebound, supporting their inclusion in future HIV-1 therapeutic options^56^.

**Fig. 7:**
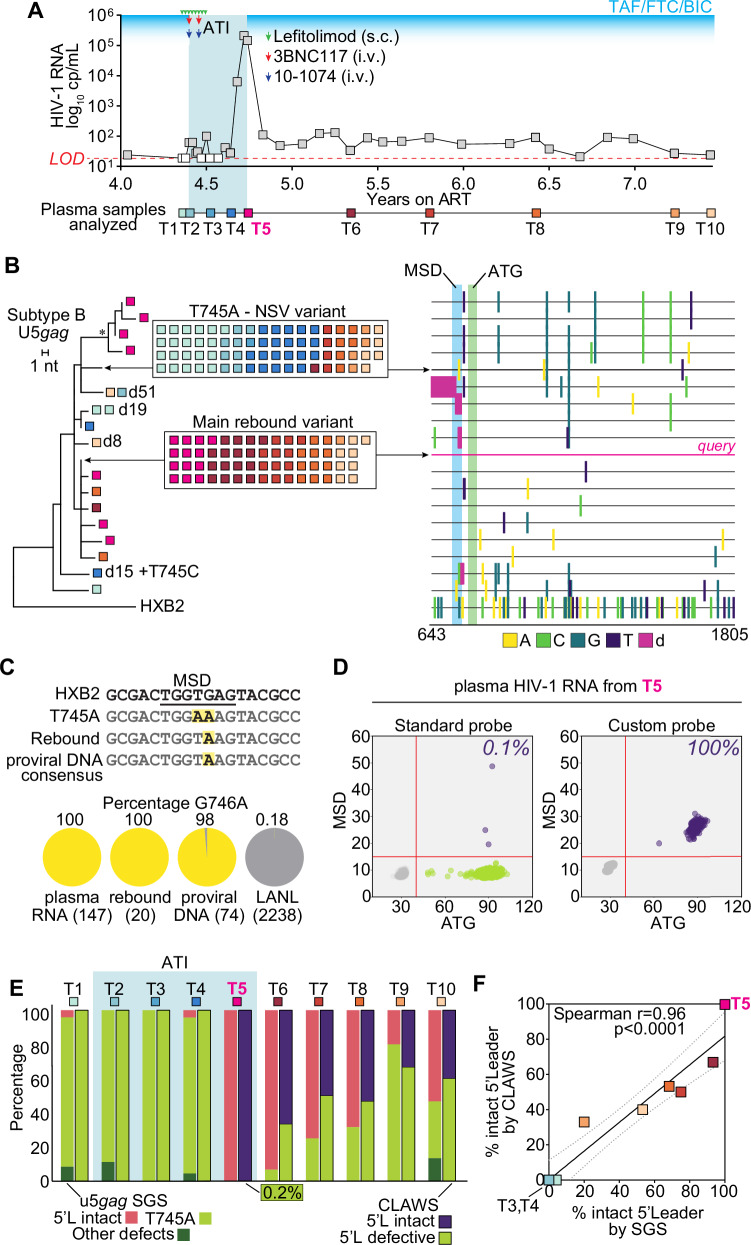
5’L defective RNA contributes to NSV in a HIV-1 cure trial participant. **A** HIV-1 RNA in plasma monitored before, during, and after experimental treatment with broadly neutralizing antibodies (bNAbs) and TLR9 agonist administered at the time of analytical treatment interruption (ATI). **B** Maximum likelihood tree and highlighter plot of 5’L*gag* plasma sequences recovered from 10 time points; MSD defects are indicated next to tree taxa; the panel on the right indicates nucleotide mismatches relative to the main viral rebound variant (query, in magenta). **C** Mutation G746A and its percentage in ID0139 sequences and the HIV-1 sequence database. **D** CLAWS 2D plots of HIV-1 RNA sampled at viral rebound; impact of the G746A mutation (left) and rescue of CALWS performance with a custom probe (right) when using the standard versus custom probe. **E** Percentage of 5’L variants by sequencing (left bars) and CLAWS (right bars). **F** Two-sided Spearman correlation analysis of intact 5’L RNA estimated by sequencing versus CLAWS (*p *< 0.0001); dotted gray lines indicate 95% confidence interval; symbols indicating timepoints T3 and T4 are behind T1 and T2.

The participant, referred to here as ID0139 (ID139 in TITAN), is a 55-year-old Caucasian male who initiated ART at diagnosis during chronic viral infection (HIV-1 RNA 800,000 copies/mL; 120 CD4⁺ T cells/μL) four years before trial enrollment. He achieved viral suppression and CD4⁺ T cell recovery (1,220 cells/μL) within four months, but developed intermittent NSV after two years on tenofovir alafenamide/emtricitabine/bictegravir. In TITAN, he received lefitolimod and bNAbs, rebounding ~18 weeks after ATI with virus resistant to 10-1074, consistent with subtherapeutic antibody levels^55^ (Fig. 7A and Supplementary Fig. S8).

We analyzed plasma from 10 time points spanning ATI initiation to 3 years of follow-up (Fig. 7A). As in the majority of PWH with NSV characterized above, 5’L*gag* sequencing revealed that NSV was driven by a predominant clone with a deleterious MSD mutation (T745A; Fig. 7B), present at 88–91% abundance. Minor variants, often with variable deletions (e.g., Δ8, Δ19, Δ51), were also detected. Conversely, plasma HIV-1 RNA at viral rebound was caused by a distinct dominant variant (80%) and other rare variants forming a related cluster, in agreement with *env* sequencing (Fig. 7B and Supplementary Fig. S8). After ART reinitiation, HIV-1 RNA rapidly fell to ~ 10² copies/mL but never became undetectable. Post-ATI, NSV was largely due to sequences that matched the rebound virus or were closely related. We posit that these sequences represent the persistence of transcriptionally active cells more recently infected during viral rebound; however, full-length proviral sequencing and matched integration-site analyses are needed to confirm this hypothesis. The T745A clonal variant also persisted, reemerging seven months after ART reintroduction (6%), and later comprising 25–80% of plasma virus.

When applying CLAWS to the same plasma samples, we found that all 5’L RNA sequences contained a G746A substitution within the MSD. Given its presence in 98% of pre-ATI proviral sequences, this is likely a transmitter–founder mutation (Fig. 7C). Although rare globally (0.18%) and not affecting replication competence (Fig. 3F–H), this change created a fatal mismatch to the MSD probe (Fig. 5A), preventing CLAWS from detecting intact 5’L RNA. We therefore designed a custom MSD probe, which restored detection of intact 5′L RNA (Fig. 7D). CLAWS estimates closely mirrored sequencing data (Fig. 6E): no intact RNA at baseline or during ATI, intact RNA during rebound, and mixed intact/defective RNA after ART reintroduction (mean 56% intact; range 33–67%, Fig. 7E). CLAWS also detected trace defective RNA (0.2%) at the peak of viral rebound, consistent with the persistence of the T745A variants, comparable to pre-rebound levels ( ~ 10^2^ copies/mL). Intactness estimates by CLAWS strongly correlated with sequencing (Spearman r = 0.96, p < 0.0001, Fig. 7F). While CLAWS cannot resolve specific viral variants or 5’L defect types, its speed, scalability, and lower cost make it an effective tool for investigating low-level HIV-1 RNA in cure-related trials.

## Discussion

The drivers of NSV are complex and include both virus and host-related factors^22,24,29^. Our study demonstrates that 5′L–defective HIV-1 RNA dominates in PWH on long-term ART who experience NSV—a perplexing and challenging clinical scenario for both PWH and providers. In 31 participants from the original NSV cohort and an additional 20 participants from the validation cohort, RNA transcribed from defective proviruses accounted for a median of 95% of plasma HIV-1 RNA, firmly establishing their central role in persistent viremia. These defects consisted of discrete deletions and point mutations tightly clustered around the MSD. Because the MSD is critical for U1 spliceosome recognition, even single-nucleotide changes disrupted RNA splicing, abolishing viral replication capacity. Nonetheless, transcription from these defective proviruses supports ongoing RNA production and viral particle release. Prior studies, limited by small sample size, left the true contribution of defective proviruses unresolved^15,23,24^. Here, in a markedly larger cohort, we demonstrate that production of RNA from 5′L-defective proviruses provides a mechanistic explanation for detectable viremia despite otherwise effective ART.

Using CLAWS, we detected 5′L-defective RNA as early as the second phase of viremia decay (∼ 30 days after ART initiation). Over time, these sequences progressively enriched, constituting >95% of plasma RNA in PWH who have been on ART for more than two decades. This pattern supports the concept that the enrichment of 5′L defective RNA in plasma is the result of progressive transformation of the proviral landscape, in agreement with recent studies showing that the reservoir is shaped over time by selective pressures^57,58^. Moreover, plasma sequences typically derived from only a few predominant clones, further underscoring the role of clonal expansion in sustaining both the reservoir and NSV^59^.

We have previously shown that 5’L defects reduce spliced mRNA transcripts, causing a significant decrease in Envelope expression on the surface of infected cells and incorporation into viral particles^15^. Thus, these proviruses may be spared from negative selection due to cytopathic effects and immune clearance, such as antibody-dependent phagocytosis (ADPC) and cytotoxicity (ADCC), contributing to positive selection over time^58^. Based on the limited near full-length HIV-1 RNA sequencing data, a similar enrichment of variants driving NSV that are exclusively deleted in *env* has not been observed^24,60^; thus, it is likely that 5’L-defects provide additional survival advantages beyond the reduced Envelope expression^61,62^. Recent work showed that 5’L defective proviruses retain expression of Nef^14,63^, which favors immune evasion by CD4 and HLA-I downregulation^64,65^, and reduces activation-induced cell death^66^, promoting overall survival. Lastly, whether the lack of Envelope incorporation affects HIV-1 viral particle clearance, extending their half-life in blood, remains unknown.

Together, these features suggest an alternative persistence model: while intact proviruses survive by transcriptional silence, due to the lower inducibility imposed by their site of integration^67^ (e.g., gene deserts, zinc finger genes), 5’L-defective proviruses evade clearance despite robust viral gene expression, creating a pool of transcriptionally active but non-infectious genomes. Future studies should dissect the mechanisms causing differential selection pressures, as they may reveal vulnerabilities of cells carrying intact and defective proviruses that could be exploited for reservoir elimination strategies. In addition, the relative contribution of intact versus defective proviruses to HIV-1 pathogenesis remains uncertain. Recent studies have shown that detection of soluble gp120 (sgp120), a mediator or bystander CD4 + T cell death and monocyte activation, is associated with lower CD4 counts and elevated IL-6 in PWH receiving effective ART^68^. These findings raise the possibility that both intact and 5′L–defective proviruses —despite altered RNA splicing— may contribute to residual sgp120 expression and ongoing immune dysregulation. Future studies are needed to define the extent to which each proviral class drives this persistent inflammatory state.

Our findings have several clinical and translational implications. First, periods of detectable viral load in adherent PWH should not always be interpreted as evidence of replication (i.e., de novo infection events), but rather virus released from defective proviruses. Indeed, among 47 individuals on long-term ART, only six had NSV caused solely by 5′L-intact RNA. Second, the striking predominance of 5′L-defective RNA in plasma challenges the notion that residual viremia reflects reservoir size and supports the observation that proviruses contributing to residual viremia before treatment interruption rarely correspond to viral rebound variants^69,70^. These findings have implications for interpreting ultrasensitive HIV-1 RNA assays to monitor the impact of experimental interventions, particularly in HIV-1 cure studies that rely on latency reversal or immune-mediated clearance, since these interventions may differentially affect intact versus defective proviruses^71^.

To enable rapid, scalable profiling of 5′L RNA intactness, we developed CLAWS, a dPCR assay that accurately quantifies intact and defective 5′L RNA. CLAWS recapitulated sequencing-based estimates, detected even low-abundance defective RNA shortly after ART initiation and during analytical treatment interruptions (ATIs), and offers a faster, cost-effective, practical tool for both clinical assessment of persistent viremia and HIV-1 cure-related trials. Although developed on a single dPCR platform (QIAcuity, Qiagen), CLAWS can be readily adapted to other systems, facilitating broad adoption. Given the widespread use of dPCR in both research and clinical labs, we think that CLAWS will become an accessible tool for PWH and care providers to improve viral load monitoring and ART management. Rapidly confirming that NSV is caused by defective proviruses can prompt ART de-intensification and mitigate anxiety, reducing concerns about virological failure and transmission. Indeed, the participants in our study widely shared significant feelings of relief with investigators attributable to their deeper understanding of viral load, validation of their consistent adherence to ART (in contrast to misguided concerns of nonadherence from some providers), avoidance of unnecessary ART modifications, and increased confidence in preventing transmission to sexual partners.

We have also shown that CLAWS can be applied to understand viral load dynamics during HIV-1 cure trials. In the last few years, experimental interventions with bNAbs resulted in ATIs with delayed or no viral rebound, often characterized by extended periods of fluctuating low-level viremia of unclear significance^72^. CLAWS has the potential to guide and expand participant selection, viral load monitoring, and better interpretation of curative intervention. Thus, future efforts will focus on increasing CLAWS’ throughput to make it even more suitable for trials with large sample sizes.

Our study has limitations. We lacked prolonged longitudinal sampling, precluding direct observation of 5’L defective RNA intra-host emergence and dynamics over time on ART. Participants were selected for persistent or intermittent viremia above 20 copies/mL, so generalizability to individuals with lower residual viremia remains untested. In its current version, CLAWS has a 95% limit of detection of 9 copies when starting from 1 mL of plasma, suggesting that it is well positioned to achieve single-copy resolution upon further optimization and by assaying larger volumes of plasma^73^. Future studies applying CLAWS longitudinally and in large cohorts of PWH with suppressed viremia will clarify the dynamics of 5’L-defective proviruses and their impact on HIV-1 persistence in all people on ART. In addition, all but two of our study participants carried subtype B virus; although CLAWS targets conserved regions, validation across non-B subtypes and circulating recombinant forms is needed before global implementation. Although straightforward and of easier application, CLAWS’ digital readout precludes recovering the exact 5’L sequence. To address this limitation, future studies could develop a highly sensitive, quantitation-preserving, sequencing method that introduces unique molecular identifiers to the CLAWS amplicon. Lastly, although our results show that 5’L-defects are a major source of NSV, proviruses with an intact 5’L but carrying small defects elsewhere in the genome can also contribute to rare cases of persistent viremia, as recently reported by our group and others^60,74^.

In summary, our results establish 5′L-defective proviruses as the major source of NSV and introduce CLAWS as a practical tool for dissecting persistent viremia. Beyond clarifying mechanisms of HIV-1 persistence, CLAWS provides immediate translational utility for clinical monitoring and HIV-1 cure research.

## Methods

### Ethics statement

The research in this study complies with all relevant ethical regulations. The Johns Hopkins University and University of Toronto Institutional Review Boards approved this study. The study participants provided written informed consent before enrollment. ID0572 and VC0001 provided written consent to an Institutional Review Board-approved study from the laboratory of Dr. Cécile Tremblay at the Center Hospitalier de l’Université de Montréal (CHUM), Canada. Participant ID0139 provided written informed consent to be enrolled in the TITAN trial and the exploratory analyses presented here. The primary outcomes of the TITAN trial (ClinicalTrials.gov ID NCT03837756) have been published previously^55^. Participants VC1001, VC1002, VC1003, VC1004, VC1005, VC1006, provided written consent to an Institutional Review Board-approved study (Dr. Wissam El Atrouni, The University of Kansas). Participants VC2001 and VC2002 provided written consent to an Institutional Review Board-approved study (Dr. Beverly Sha, Rush University). Participants from the NIH provided written informed consent for research and for sharing samples in protocols 97-I-0082, 95-I-0027, and 08-I-0221, and were approved by the National Institute of Allergy and Infectious Diseases (NIAID) Institutional Review Board (FWA00005897).

### Study participants

Study participants were referred by their HIV-1 care providers at Johns Hopkins University’s Bartlett Specialty Clinic (Baltimore, MD, USA), Maple Leaf Medical Clinic and the University of Toronto (Toronto, Ontario, Canada), the Center Hospitalier Universitaire du Montreal (CHUM) (Montreal, Quebec, Canada), the Aarhus University Hospital (Aarhus, Denmark), Sierra Infectious Diseases Clinic (Reno, NV, USA). Additional participants where involved for the validation cohort from Johns Hopkins University’s Bartlett Specialty Clinic (Baltimore, MD, USA), Maple Leaf Medical Clinic and the University of Toronto (Toronto, Ontario, Canada), the Centre Hospitalier Universitaire du Montreal (CHUM) (Montreal, Quebec, Canada), The University of Kansas Medical Center (Kansas City, KS), and Rush University Medical Center (Chicago, IL). Peripheral blood samples were collected at one or multiple timepoints (2022-2025). For ID0139, samples were obtained during and as a follow-up to the TITAN clinical trial between 2021 and 2024^55^. Longitudinal samples collected before and after ART introduction (AVBIO) were obtained from the National Institutes of Health (1997-2001).

### Sample collection and processing

Blood was collected from peripheral phlebotomy into vials containing EDTA and immediately processed as previously described^73^. Briefly, blood was spun at 400 × *g* for 10 min, and the isolated plasma was spun a second time at 1350 g for 15 min, then frozen in 1-1.8 mL aliquots and stored in liquid nitrogen. Peripheral blood mononuclear cells (PBMCs) were isolated from either whole blood or leukapheresis by density gradient separation as previously described^6^. For some participants, plasma was collected from leukapheresis samples.

### Study of HIV-1 RNA sequences in plasma

Once thawed, 1-1.5 mL of plasma were aliquoted into separate microcentrifuge tubes and centrifuged at 4 °C for 15 min at 2700g. Next, plasma was aliquoted into new microcentrifuge tubes, taking care to leave behind any visible cellular debris. The tubes were then spun at 4 °C for 2 h at 21,000 × *g*. RNA was extracted from virion pellets as recently described^75^. RNA was resuspended in 13 μL of 5 mM Tris HCl and left on ice for 15’ to resuspend, and then used for cDNA synthesis using Induro Reverse Transcriptase (New England Biolabs) and the reverse outer primers of the 5’L*gag* and *p6*RT amplicons (u5gagRO TGACATGCTGTCATCATYTCYTC and 3500 CTATYAAGTCTTTTGATGGGTCATAA, respectively). Synthesis of cDNA was performed as follows: a mix of 12.5 μL of RNA, dNTPs (1 μL,10 mM), and reverse primer (0.21 μL, 50 μM) undergoes denaturation at 65 °C for 5 min, then is immediately quenched on ice for 1 min. A second mix with Induro RT enzyme (1 μL), 5x buffer (5 μL) and RNAse inhibitor (0.25 μL, 40 μ/μL) is added before incubation at 55 °C for 30 min, followed by 95 °C for 1 min, and held at 4 °C.

### Single genome sequencing (SGS) of plasma HIV-1 RNA

cDNA was amplified by outer PCR and by nested PCR, with previously published primers^15^. Outer PCR was conducted using VeriFi Polymerase (PCR Biosystems) due to its higher tolerance to inhibitors. The following parameters were used for outer PCRs: 1 cycle of 95 °C for 2 min, 45 cycles of 95 °C for 15 s, 53 °C for 15 s, 72 °C for 1 min and 15 s, 1 cycle of 72 °C for 3 min, and held at 4 °C. Nested PCR was conducted using Platinum Taq High Fidelity DNA Polymerase (Thermo Fisher Scientific). The following parameters were used for nested PCRs: 1 cycle of 94 °C for 2 min, 45 cycles of 94 °C for 30 s, 55 °C for 30 s, 72 °C for 2 min, 1 cycle of 72 °C for 3 min, and held at 4 °C.

### CD4+ T cells Isolation and HIV-1 DNA SGS

CD4^+^ T cells were isolated from PBMCs using EasySep Human CD4^+^ T Cell Isolation Kit (StemCell Technologies) per the manufacturer’s instructions. Extraction of gDNA and HIV-1 DNA SGS was done as previously described^15,76^. The Intact Proviral DNA Assay was performed as previously described^17^.

### Analyses of HIV-1 sequences

Electrophoresis was conducted on nested PCR products to identify positive reactions. PCR products yielding single bands were purified and sequences by Sanger sequencing (Genewiz Azenta). Sanger sequences were assembled in contigs and subjected to quality control with the software Geneious, then aligned and manually inspected with Bioedit. The analysis of drug resistance mutations was based on the 2025 update from the International AIDS Society Drug Resistance Mutations Group^77^. The phylogenetic trees in Figs. 2A, 6B, and Supplementary Fig. S2A were generated by Maximum Likelihood with a GTR + G + I substitution model, identified with MEGA v7.0, and 1000 bootstrap replications. Phylogenetic trees from Fig. 5B and S6 were generated by Maximum likelihood with the HKY + G + I model, identified as the most accurate model. HIV-1 RNA sequences used for the reference dataset were collected from the Los Alamos National Laboratory (LANL) HIV Database. Sequences used were recovered from plasma RNA, one per individual, without filtering per subtype. Analysis of HIV-1 sequence entropy for the CLAWS assay primer design was performed using the Entropy-one tool from the LANL HIV sequence database. Additional analyses of sequences were conducted as previously described^15^.

### Testing the impact of single nucleotide mutations in the MSD

Site-directed mutagenesis (New England Biolabs) was used to introduce mutations into the MSD of a pNL4-3 plasmid. Plasmid were confirmed by colony PCR and Sanger sequencing. Full-length sequences of the final plasmids were confirmed by long-read sequencing by Oxford Nanopore (Plasmidsaurus). 293 T cells, obtained through BEI Resources, NIAID, NIH (NR-9313), were then transfected using polyethylenimine (PEI) with 5ug of PAdvantage (Promega) and 30ug of plasmids containing one of the following mutations: G744A, T745A, G746A, G748A. Wildtype and Δ*env* pNL4-3 were also used as controls. After 72 h, the supernatant was spun and concentrated using the Lenti-X Concentrator (Takara). Virus was quantified by p24 ELISA (Revvity Alliance HIV-1 p24 Antigen ELISA Kit). Infectivity was tested by spinoculation of CD4^+^ T cells from a healthy donor. Cells were activated for 3 days in the presence of anti-CD3/CD28 coated beads (Thermo Fisher) and plated at 1 million cells per 100 μl of RPMI media with 10% Fetal Bovine Serum (R10) in a V-bottom 96 well plate. We added 10 ng p24 of each viral stock and spun at 1200 × *g* for 2 h at 25 °C. Each condition was tested in triplicate wells. The plate was then incubated at 37 °C for 5 h, washed five times with cold media, and cells were moved to a 24-well plate at 1 million cells/mL in R10 with 100 μI of IL-2/mL and antimicrobial solution (Sigma Aldrich). After 1 h, 200 μL of supernatant was collected for the baseline time point. We then collected 200 μL of supernatant and replaced fresh media at 12, 24, 48, 72, and 196 h. P24 was measured by ELISA (Revvity Alliance HIV-1 p24 Antigen ELISA Kit). The plasmids generated for these experiments are available upon request to the corresponding author.

### CLAWS assay (Capturing 5’Leader Anomalies Without Sequencing)

CLAWS is based on a single amplicon and two probes, designed based on regions the highest sequence conservation. To maximize specificity in distinguishing intact from defective 5’L RNA, and to minimize assay failure due to polymorphisms, the probes are only 10 nucleotides long and contain locked nucleic acids (LNAs, Integrated DNA Technologies), which increase melting temperature. Primers and probes were used at a final concentration of 450 nM and 125 nM, respectively; sequences for primers and probes can be found in supplementary Table S2. For CLAWS, RNA was isolated and used for cDNA synthesis as described above, but the CLAWS reverse primer was used during RT. CLAWS was developed for the QIAcuity dPCR system (Qiagen), using the 24 well nanoplates (24,000 partitions) and the following reagents for each reaction (40 μL total): 10 μL of QIAcuity Probe Mastermix, 1 μL of 40x CLAWS primer/probe mix, 19 μL PCR-grade water, and 10 μL template (cDNA or gDNA). The following parameters were used for the dPCR: 1 cycle of 95 °C for 2 min followed by 45 cycles of 95 °C for 15 s, and 61 °C for 40 s. Each cDNA sample was tested in two dPCR reactions.

### CLAWS calculations and visualization

Copies of MSD and ATG per μL of dPCR reaction are normalized based on total reaction volume (40 μL), cDNA input (10 μL), and cDNA reaction volume (20 μL) and RNA input (12.5 μL). Then copies/mL of plasma are calculated based on the starting volume of plasma extracted. Results from two technical dPCR replicates are averaged, and if multiple aliquots are tested, the average values are reported. The percentage of intact MSD RNA is calculated based on the number of double positive (dp) partitions for the MSD and ATG probes and the number single positive (sp) for the ATG probe ([dp ^MSD, ATG^/(dp^MSD, ATG^ + sp^ATG^)]*100, see Fig. 4C). To visualize dPCR raw data, we exported the raw data csv files (“RFU values, compact”) and used them as input for the R package Tidyplots^78^. Invalid partitions were filtered, and the files for green and yellow fluorescence were merged. Due to the high number of partitions per file ( > 25,000), we reduced image size by randomly subsampling negative partitions (*n* = 500).

### CLAWS validation experiments

CLAWS optimization and specificity were assessed with a panel of 500 nucleotide synthetic double-stranded DNAs (Gblocks, Integrated DNA Technologies) containing the HXB2 reference sequence, in which we introduced defects recovered from participants with NSV. The following deletions were tested: Δ2, Δ3, Δ17, Δ21, Δ22, Δ33, Δ48, Δ97. All of the deletions, except for Δ97, were detected by CLAWS as single positive in ATG and therefore having a mutation in their MSD. The Δ97 deletion was double negative, as this deletion includes both the MSD and ATG probes and the forward primer. The following point mutations were tested: G744A, G744C, G744T, T745A, T745C, T745G, G746A, G746T, and G748A (see Supplementary Fig. S5A). To further test CLAWS specificity and accuracy in detecting the percentage of intact 5’L RNA, we diluted stocks of wildtype and T745A NL4-3 in the plasma of pooled healthy donors to achieve ~ 50cp/mL. Diluted viruses were then mixed at different ratios (Fig. 4F) to generate 1 mL replicate aliquots. Eight to nine aliquots for each mix were used to isolate RNA, generate cDNA, and tested by CLAWS as described above.

CLAWS limit of detection and linearity were tested by diluting NL4-3 wildtype virus to 1:1 × 10^6^cp/mL with 1 x DMEM. Next, 100 μL of DNAse I Solution (StemCell Technologies) were added, and samples were incubated at RT for 30 minutes to eliminate carryover of plasmid DNA. Further 3-fold serial dilutions in plasma from pooled healthy donors (Innovative Research, lots 53961 and 56071) were made (1:81, 1:243, 1:729, 1:2,187, 1:6,561, 1:19,683) and distributed over 10 aliquots of 1 mL each. These samples were used as input for CLAWS, and the 95% limit of detection was calculated by probit analysis using the SAS software version 9.4 (SAS Institute Inc.). To further validate CLAWS sensitivity, we tested controls from the Virology Quality Assurance (VQA) program (lot 24129022) at Duke University. We tested 10 aliquots expected to contain 20cp/mL and 10 aliquots containing 0cp/mL (1.8 mL each). CLAWS’ limit of blank (LOB) was estimated by screening 12 mL of HIV-1 negative pooled plasma (Innovative Research, lots 53961 and 56071). None of the reactions resulted in positive for HIV-1 RNA (LOB = 0).

### Application of CLAWS on clinical samples collected before and after ART introduction

The clinical samples obtained before ART were diluted in 1x TBS (Quality Biological) to contain 10,000cp/mL or less. To reduce the frequency of positive partitions and co-localization of multiple RNA copies per partition, RNA was resuspended in 80 μl of 5 mM tris HCl. The cDNA was generated and then diluted with 5 mM Tris HCl to a final volume of 60 μl. Samples collected during the second phase decay of viremia were processed and tested by CLAWS as those from individuals with NSV.

### Statistical analyses

Analyses were performed with GraphPad Prism v8.0, including descriptive statistics and tests for normality (Shapiro-Wilk and Kolmogorov-Smirnov tests) to determine which test to use in downstream analyses. Differences between the two groups were tested by parametric and non-parametric *t* tests (i.e., Mann-Whitney). In case of paired data between two groups, we used the Wilcoxon paired rank test. The chi-square test was used to compare the frequency of MSD point mutations between the HIV Database and the NSV dataset. We used Spearman’s rank-order correlation test for comparing the estimate of intact 5’L RNA obtained by sequencing and CLAWS, and for the analysis of CLAWS limiting dilution data. A *p*-value of < 0.05 was considered significant unless otherwise stated. The significance of maximum-likelihood phylogenetic tree nodes was tested by the bootstrapping method (500 replications) implemented in MEGA v7.0.

### Reporting summary

Further information on research design is available in the Nature Portfolio Reporting Summary linked to this article.

## Supplementary information

Supplementary information

Reporting summary

Transparent Peer Review file

## Source data

Source Data File

## Data Availability

All data associated with this study are in the main text, supplementary materials; source data are in the Source Data files, provided with this paper. HIV-1 sequences are available on GenBank (OQ092462-OQ92467, PZ328790-PZ329638, PZ329639-PZ329991, PV927540-PV927951, PZ068438-PZ068920). Source data are provided in this paper.

## Code availability

The R script for the Tidyplot package used to plot dPCR data is publicly available at: https://github.com/FRSimonetti/CLAWS_dPCR_plotting, archived at Zenodo 10.5281/zenodo.20077757.
