# Supplementary material for: 5′ leader defects drive persistent HIV-1 viremia on long-term ART
Source: Nat Commun. 2026 Jun 8;17:4725. doi: 10.1038/s41467-026-73475-5 (PMC13246943; doi:10.1038/s41467-026-73475-5)
Supplement: Supplementary file 1 — Supplementary information [file 41467_2026_73475_MOESM1_ESM.pdf]

Supplementary Information

5′ Leader Defects Drive Persistent HIV-1 Viremia on Long-Term ART

TABLE OF CONTENTS

Supplementary Figures:

Figure S1. HIV-1 RNA in plasma over time in participants with nonsuppressible viremia, related to Fig.1.

Figure S2. Analysis of P6RT region from HIV-1 RNA in plasma shows predominant variants and lack of drug resistance to concurrent protease or reverse transcriptase inhibitors, related to Fig. 1.

Figure S3. Distribution of intact and defective 5'Leader RNA variants in PWH with NSV, related to Figure 2.

Figure S4. Proviruses from $CD4^+$ T cells are a distinct population than HIV-1 RNA variants in plasma, related to Fig. 2.

Figure S5. Validation of CLAWS specificity and sensitivity, related to Fig. 4 and 5.

Figure S6. Characterization of a validation cohort of individuals with NSV, related to Fig. 5.

Figure S7. 5'L defective RNA can be detected in plasma during second phase decay of viremia, related to Fig. 6.

Figure S8. Proviruses with 5'Leader defects have a frequency comparable to intact proviruses measured by IPDA (intact proviral DNA assay), related to Fig. 6.

Figure S9. HIV-1 variants causing rebound in ID0139 are distinct from the proviral population obtained before analytical treatment interruption, related to Fig. 7.

Supplementary Tables:

Table S1. Participant characteristics – original NSV cohort.

Table S2. Participant characteristics – validation cohort.

Table S3. CLAWS assays oligos.

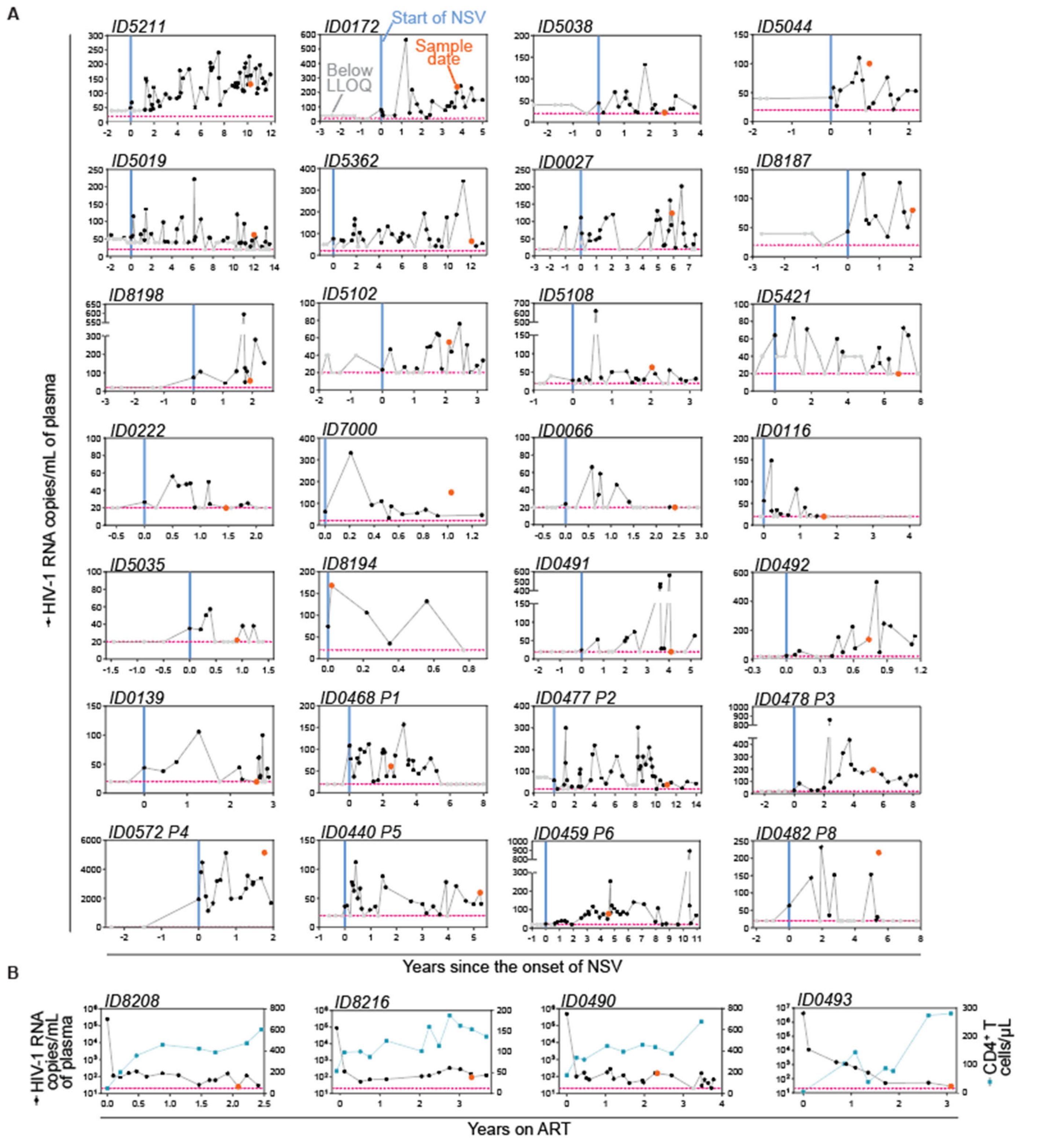

Figure S1. HIV-1 RNA in plasma over time in participants with nonsuppressible viremia, related to Fig.1. (**A**) HIV-1 RNA measurements of the 28 participants on long-term ART; grey symbols indicate values below the lower limit of quantification (LLOQ); orange symbols indicate sampling date; when HIV-1 RNA data is not available on the date of blood collection, the symbol is not connected to the other data points. (**B**) HIV-1 RNA and $CD4^+$ T cell counts of four participants who never achieved suppression since the beginning of ART.

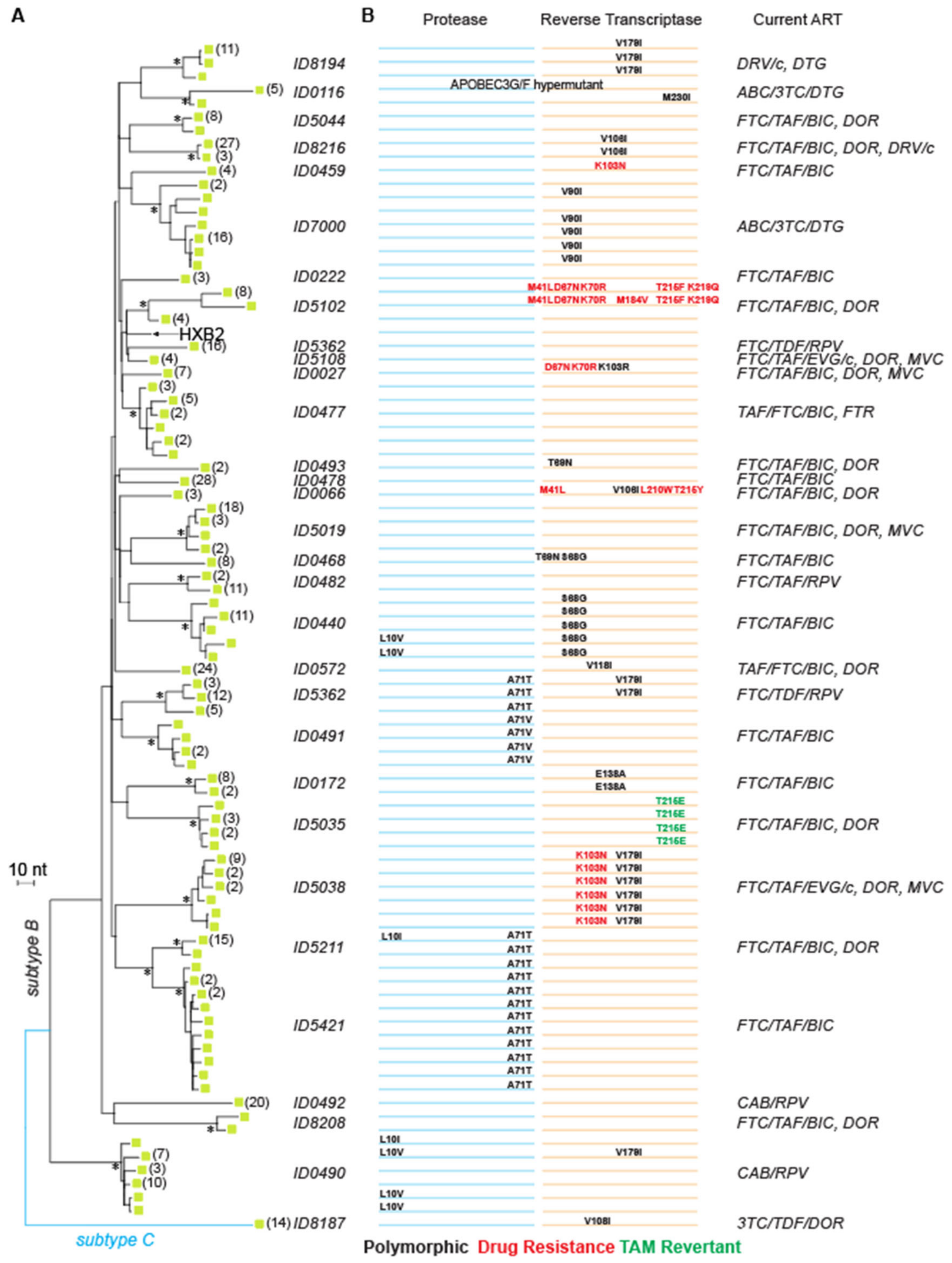

Figure S2. Analysis of *p6*RT region from HIV-1 RNA in plasma shows predominant variants and lack of drug resistance to concurrent protease or reverse transcriptase inhibitors, related to Fig. 2. (**A**) Maximum likelihood phylogenetic tree of *p6*RT unique sequences from 31 participants, using a GTR+G+I substitution model; the tree is rooted to one subtype C variants from participant OM8187; nodes with bootstrapping values above 85 are indicated with a star symbol; the number in parenthesis indicates the number of identical sequences recovered. (**B**) Analysis of amino acid mutations associated with drug resistance; mutations in black are polymorphic with no impact to drug susceptibility when in isolation, those indicated in red confer drug resistance, those in green are revertants of thymidine associated mutations (TAMs); current ART is indicated on the right.

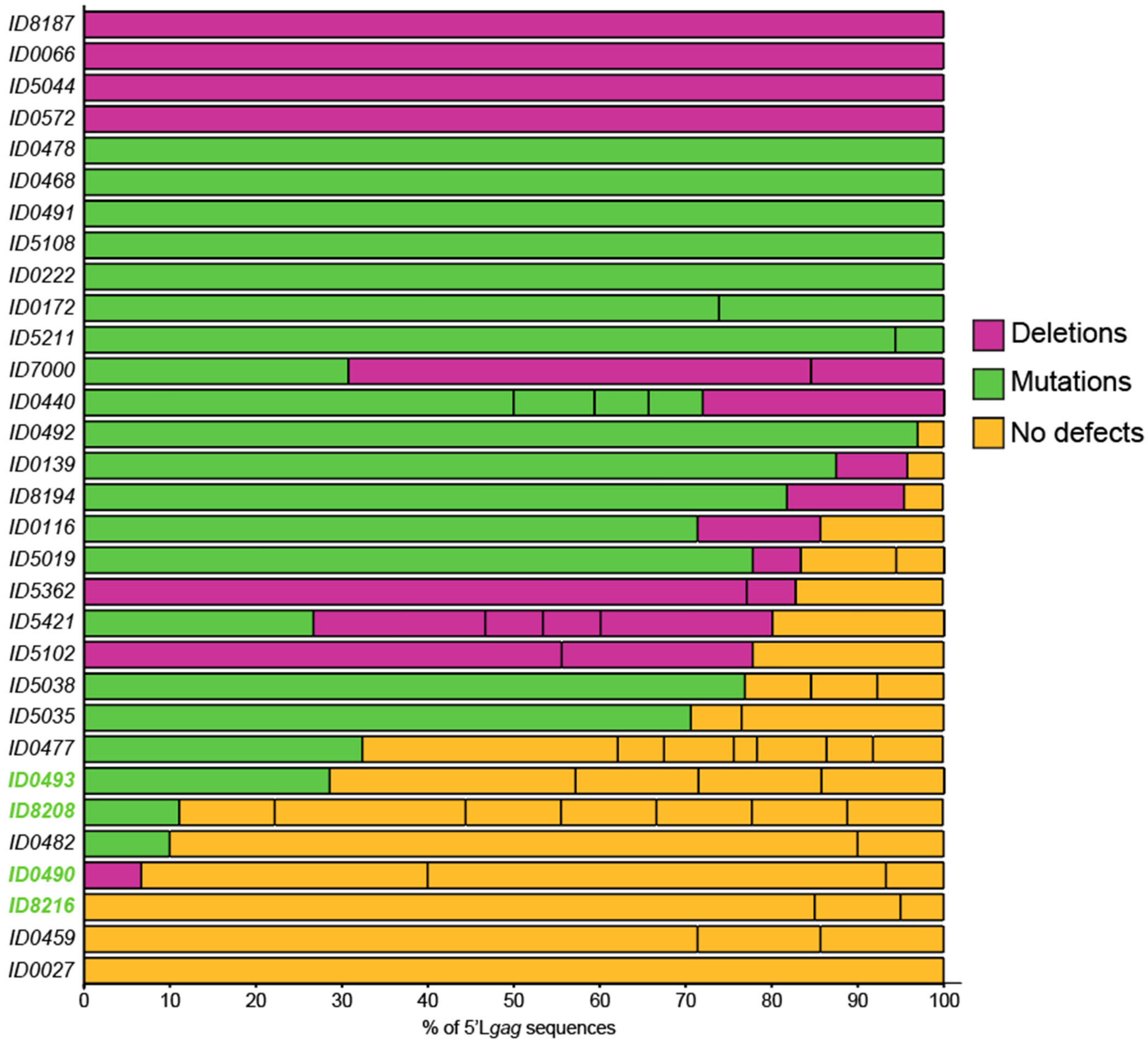

Figure S3. Distribution of intact and defective 5'Leader RNA variants in PWH with NSV, related to Fig. 2. Each horizontal stacked bar indicates the percentage of sequences belonging to a certain variant; 5'Leader variants with deletions, mutations in the MSD, or no defects, are color coded as in the legend on the right. Participants are sorted based on the overall percentage of intact variants in plasma (from 0 to 100%); Participants who have NSV after short-term ART are indicated in green as in Fig. 2A and H.

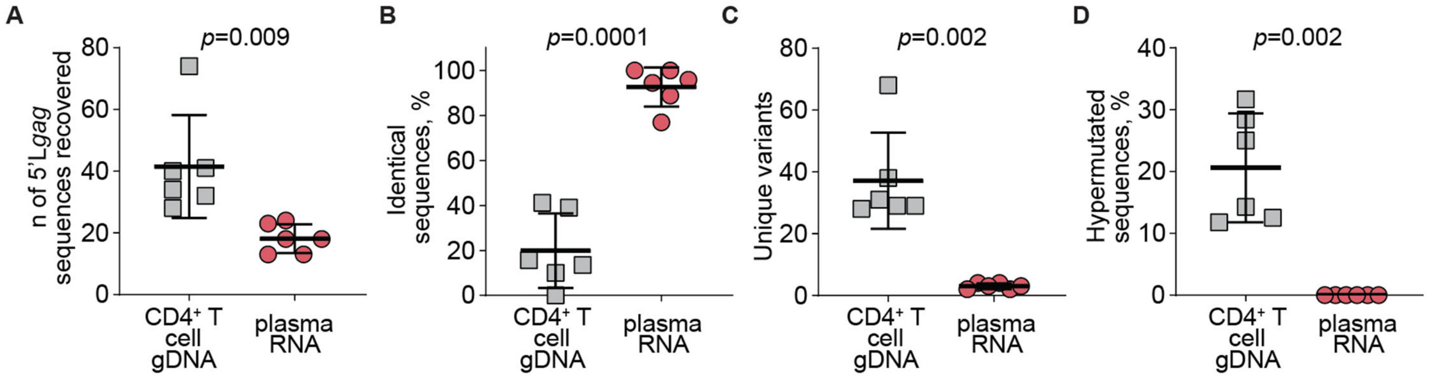

Figure S4. Proviruses from CD4$^+$ T cells are a distinct population than HIV-1 RNA variants in plasma, related to Fig. 2. (**A**) Number of 5'L*gag* sequences recovered from proviruses and virus in plasma; each symbol indicates a study participant (n=6). (**B**) Percentage of identical sequences. (**C**) Number of unique variants recovered. (**D**) Percentage of sequences with evidence of APOBEC3G/F-induced hypermutation. Grey squares indicate proviral DNA, circles in red indicate HIV-1 RNA sequences; horizontal bars indicate mean and standard deviation; p values obtained by parametric two-sided paired t-test.

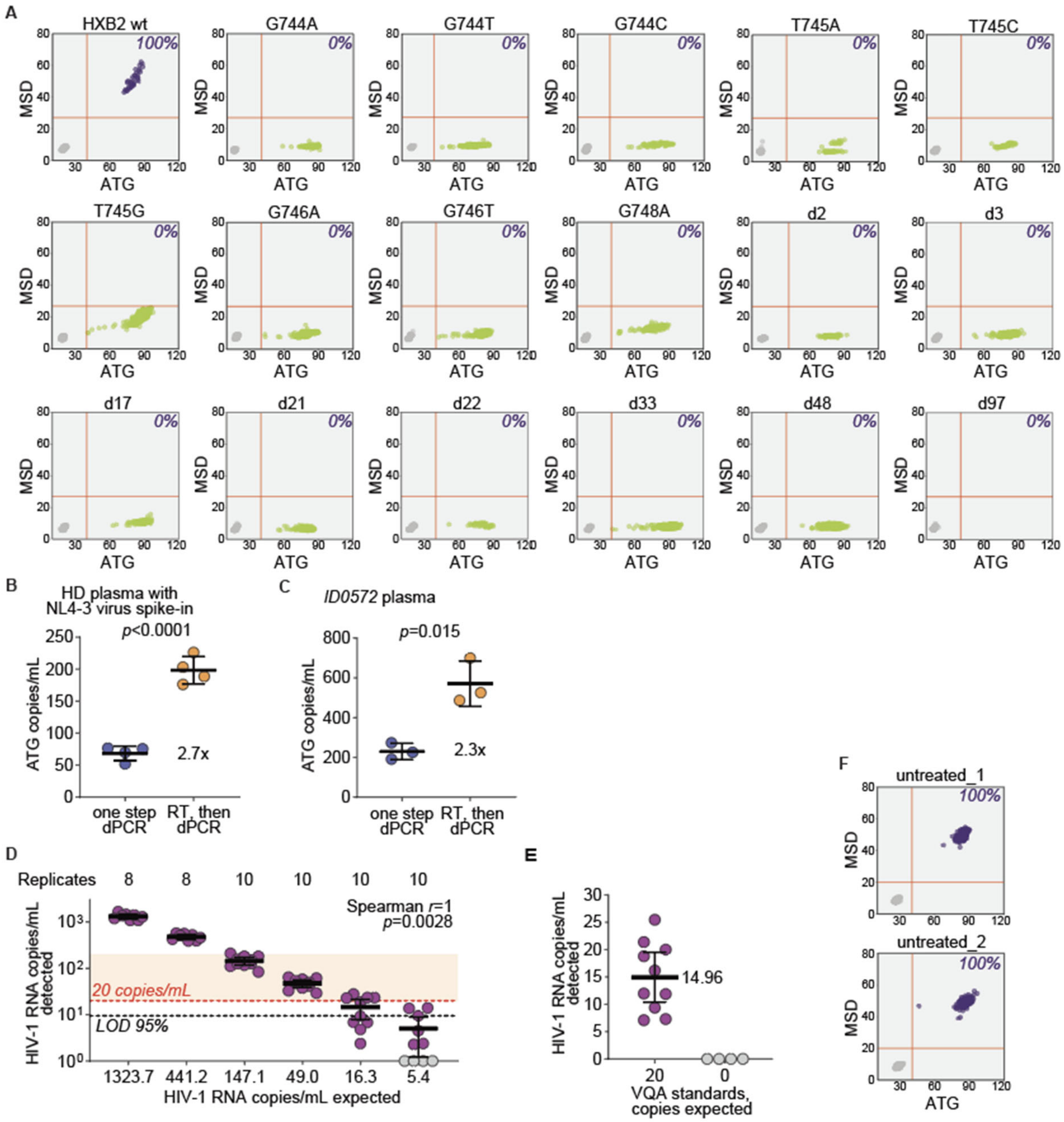

Figure S5. Validation of CLAWS' specificity and sensitivity, related to Fig. 4 and 5. (**A**) Two-dimension plots of CLAWS from synthetic double-stranded DNA; the number in the upper right corner indicates the percentage of intact 5'L; larger deletions (e.g., d97) that extend into the primer annealing regions, result in negative partitions. (**B**-**C**) Comparison of CLAWS's efficiency when using one-step dPCR versus cDNA synthesis and then dPCR; each symbol indicates a replicate aliquot of plasma (n=4 and n=3, respectively); horizontal bars indicate mean and standard deviation; p value obtained by two-sided parametric t-test. (**D**) Linearity and limit of detection of CLAWS on NL4-3 virus treated with DNAseI and spiked into pooled plasma from HIV-1 negative donors; horizontal bars indicate mean and 95% confidence intervals; grey symbols indicate HIV-1 RNA negative replicates; the dashed black line indicates the 95% limit of detection based on the probit analysis (9 copies/mL); the tan shaded area indicates the typical range of viremia in people with NSV. (**E**) Quantification of HIV-1 RNA by CLAWS of the standards from the Virology Quality Assurance program (n=20 and n=4); horizontal bars indicate mean and 95% confidence intervals. (**F**) CLAWS 2-dimension plots from two representative individuals naïve to ART.

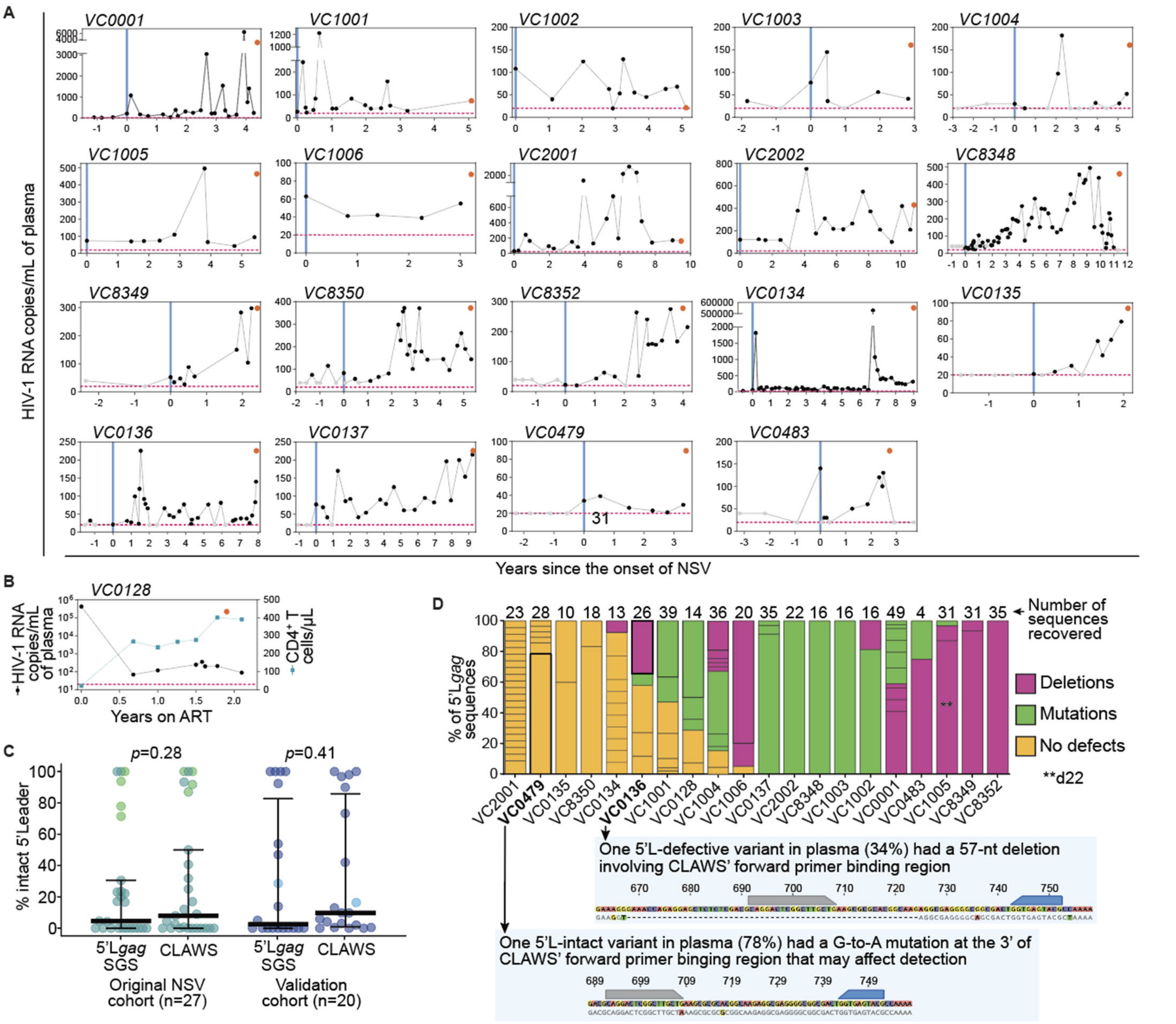

Figure S6. Characterization of a validation cohort of individuals with NSV, related to Fig. 5. (**A**) HIV-1 RNA measurements of 19 participants on long-term ART; grey symbols indicate values below the lower limit of quantification (LLOQ); orange symbols indicate sampling date; when HIV-1 RNA data is not available on the date of blood collection, the symbol is not connected to the other data points. (**B**) HIV-1 RNA and CD4^{+} T cell counts of one participant who never achieved suppression since the beginning of ART. (**C**) Percentage of 5'L-intact RNA measured by 5'L*gag* single genome sequencing and CLAWS, for both the original and the validation cohorts; each symbol indicates one study participant, with color matching Fig. 5; error bars indicate median and interquartile range; the differences between the two assays, for each group, were tested by paired non-parametric two-sided Wilcoxon test (**D**) Stacked bar plot indicating the percentage of 5'L*gag* RNA sequences belonging to a certain variant; 5'L variants with deletions, mutations in the MSD, or no defects, are color coded as in the legend on the right. Participants are sorted based on the overall percentage of intact variants in plasma (from 100 to 0%); two variants from participants VC0479 and VC0136 are described in the blue-shaded inserts, to highlight features affecting CLAWS detection.

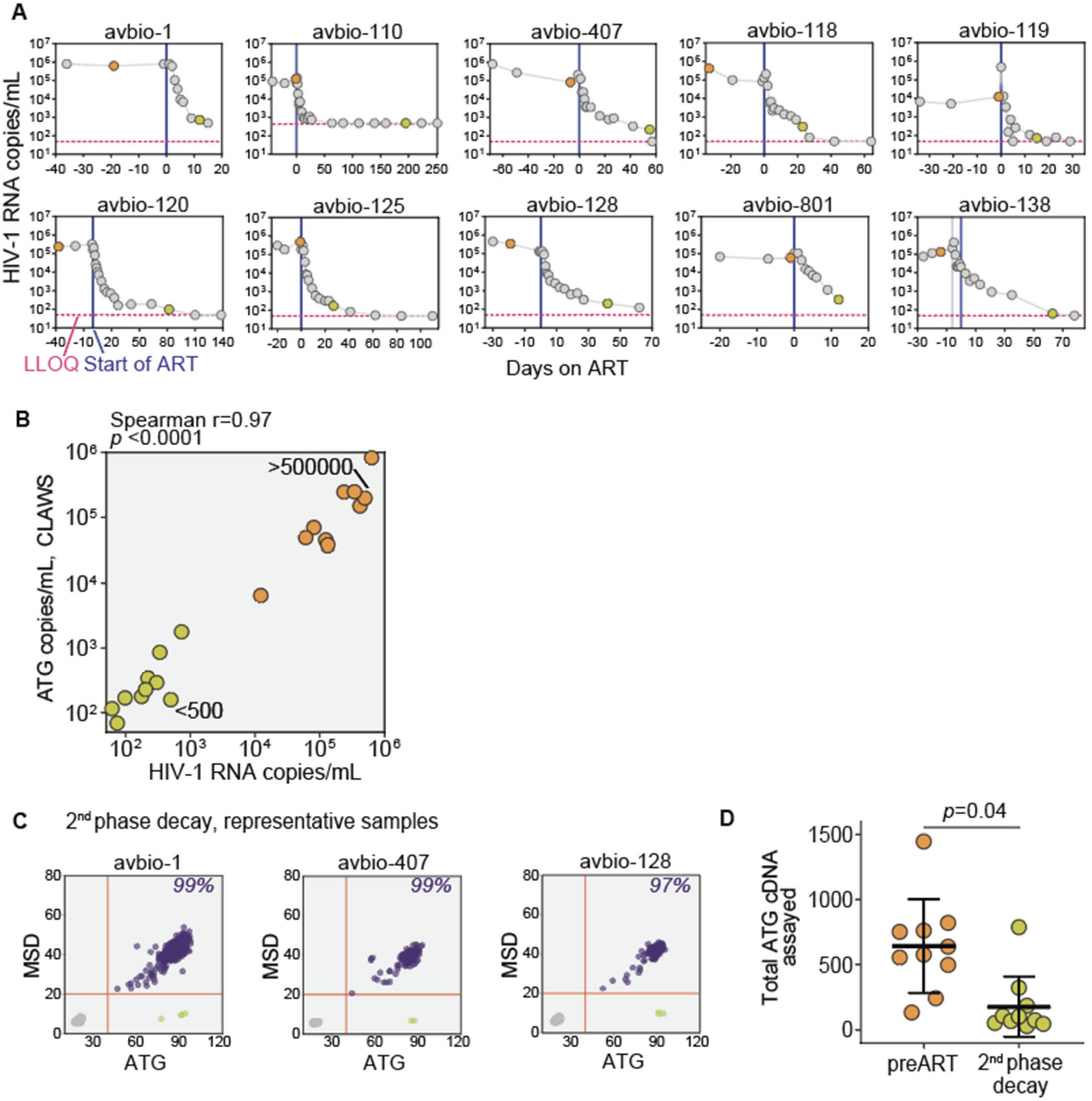

Figure S7. 5'L defective RNA can be detected in plasma during second phase decay of viremia, related to Fig. 6. (**A**) Plasma HIV-1 RNA from 10 individuals starting ART; LLOQ indicates the lower limit of quantification, which is 400/mL copies for avibio-110 and 50 copies/mL for the remaining participants; colored symbols indicate the timepoints of the samples tested by CLAWS, shown in Fig. 6. (**B**) Two-sided Spearman correlation analysis of HIV-1 RNA measured with the clinical assay versus CLAWS. (**C**) Representative 2-dimentions plots of HIV-1 RNA in plasma during second phase decay of viremia. (**D**) Number of HIV-1 cDNA molecules analyzed by CLAWS; horizontal bars indicate mean and standard deviation; p value obtained by two-sided non-parametric Wilcoxon test.

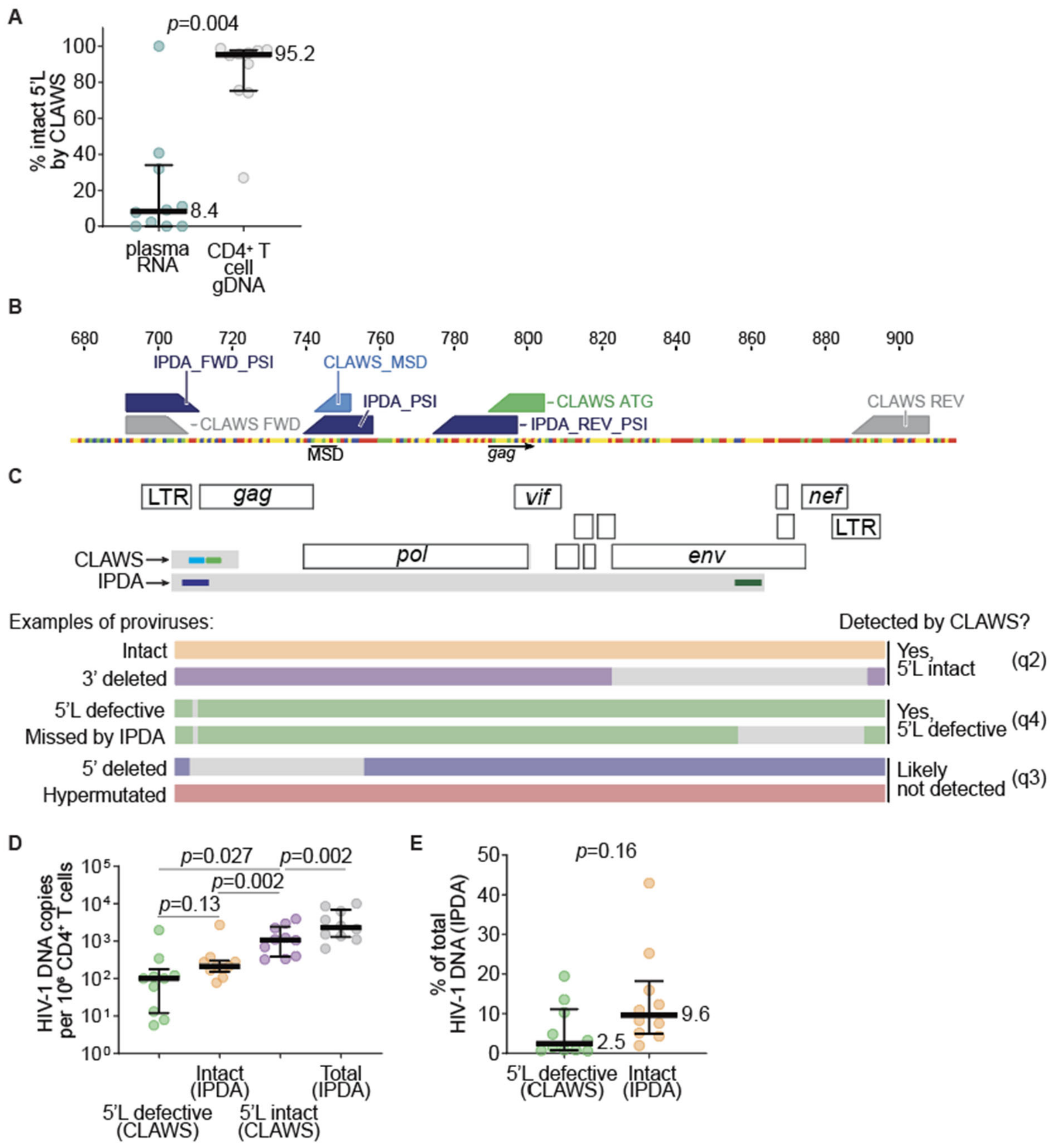

Figure S8. Proviruses with 5'Leader defects have a frequency comparable to intact proviruses measured by IPDA (intact proviral DNA assay), related to Fig. 6. (**A**) Percentage of intact 5'Leader (MSD and ATG double positive partitions based on CALWS) in plasma RNA and in CD4+ T cell-derived genomic DNA (gDNA) from 10 participants on long-term ART. (**B**) Location of primers and probes used for CLAWS and IPDA (PSI) relative to the HXB2 reference sequence. (**C**) Map of the HIV-1 genome showing the location of both CLAWS and IPDA targets; the lower panel shows example of proviruses and how they would be scored by CLAWS. (**D**) Frequency of HIV-1 DNA copies per million CD4+ T cells from the same participants in A; different types of HIV-1 DNA were compared: CLAWS was used to quantify 5'L intact (purple) and defective (green) HIV-1 DNA, while the IPDA was used to quantify intact (orange) and total (grey) HIV-1 proviruses. (**E**) Percentage of 5'L-defectve and IPDA-intact proviruses relative to total HIV-1 DNA measured by IPDA total proviruses. For **A**, **D**, and **E**, horizontal bars indicate median and interquartile range; statistical analyses were conducted by applying two-sided non-parametric paired t-test (Wilcoxon test), n=10.

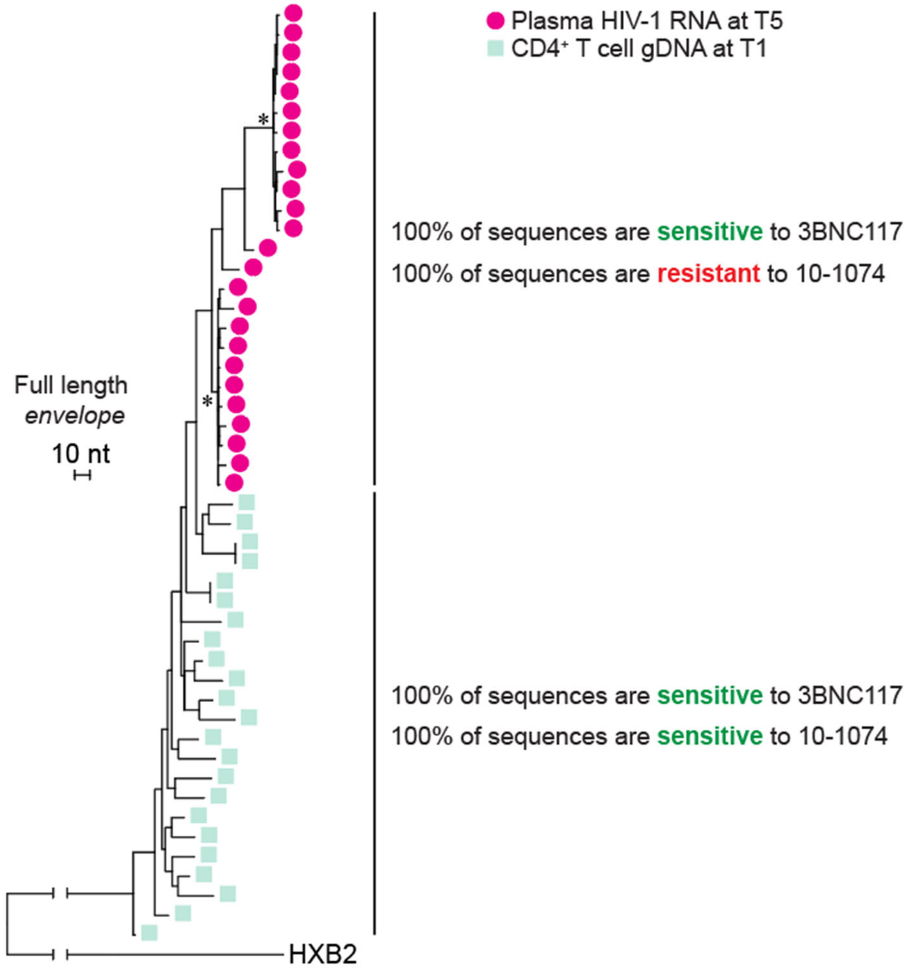

Figure S9. HIV-1 variants causing rebound in ID0139 are distinct from the proviral sequences obtained before analytical treatment interruption, related to Fig. 7. Maximum Likelihood phylogenetic tree of full-length *envelope* sequences, based on the HKY+G+I substitution model. Nodes with bootstrap values higher than 85 are indicated with star symbols; sensitivity to broadly neutralizing antibodies (bNAbs) was determined as previously described[55].

Table S1. Participant characteristics – original NSV cohort. Numbers in red indicate values above the upper limit of quantification. Outcome "resolved" refers to cases in which plasma HIV-1 RNA returned to undetectable levels for more than six months. ART, antiretroviral therapy; NSV, nonsuppressible viremia; FTC, emtricitabine; TAF, tenofovir alafenamide; TDF, tenofovir disoproxil; BIC, bictegravir; MVC, maraviroc; DOR, doravirine; ABC, abacavir; 3TC lamivudine; DRV, darunavir; c cobicistat; EVG, elvitegravir; DTG, dolutegravir; RPV, rilpivirine; FTR, fostemsavir; CAB cabotegravir. Na indicates values not available.

#	Participant ID	Sex	Race/Ethnicity	CD4+ T cells/μL, nadir	CD4+ T cells/μL, most recent	HIV-1 RNA cp/mL, Pre-ART	Years on ART	HIV-1 RNA cp/mL, average during NSV	HIV-1 RNA cp/mL, standard deviation during NSV	Years of observed NSV	NSV type	Outcome	Current ART	HIV-1 subtype based on u5gag	Previously published with participant ID (ref)
1	ID0027	Male	White/Caucasian	na	798	8675	34	69.1	45.2	7.4	intermittent	ongoing	FTC/TAF/BIC, MVC, DOR	B	
2	ID0116	Male	White/Caucasian	na	745	70637	24	39.1	33.7	1.5	intermittent	resolved	ABC/3TC/DTG	B	
3	ID0066	Male	White/Caucasian	na	946	165479	35	32.3	16.3	1.4	intermittent	resolved	FTC/TAF/BIC, DOR	B	
4	ID0172	Male	Latino/Hispanic	290	289	132604	16	133.8	107.7	5.0	continuous	ongoing	FTC/TAF/BIC,	B	
5	ID0222	Male	White/Caucasian	na	1509	na	23	31.0	13.3	1.9	intermittent	unclear	FTC/TAF/BIC,	B	
6	ID5019	Male	White/Caucasian	70	445	500000	20	47.7	32.8	13.5	intermittent	ongoing	FTC/TAF/BIC, MVC, DOR	B	
7	ID5038	Male	White/Caucasian	390	640	500000	16	42.8	26.2	3.8	continuous	ongoing	FTC//TAF/EVG/c, DOR, DRV	B	
8	ID5035	Male	Mixed	220	859	221635	16	28.9	12.1	1.4	intermittent	unclear	FTC/TAF/BIC, DOR	B	
9	ID5044	Male	White/Caucasian	380	859	189525	13	51.7	23.9	2.2	continuous	ongoing	FTC/TAF/BIC, DOR	B	
10	ID5102	Male	White/Caucasian	na	870	351786	37	33.4	17.0	3.2	intermittent	ongoing	FTC/TAF/BIC, DOR	B	
11	ID5108	Male	White/Caucasian	na	401	4284	37	53.9	114.1	3.2	intermittent	ongoing	FTC/TAF/EVG/c, MVC, DOR	B	
12	ID5211	Male	White/Caucasian	120	663	212183	22	122.7	52.6	12.0	continuous	ongoing	FTC/TAF/BIC, DOR	B	
13	ID5362	Male	White/Caucasian	270	373	500000	19	89.3	59.9	13.3	intermittent	ongoing	FTC/TDF/RPV	B	
14	ID5421	Male	White/Caucasian	162	402	313754	13	39.5	18.7	7.3	intermittent	resolved	FTC/TAF/BIC	B	
15	ID7000	Male	Black/African Descent	na	1116	500000	29	88.7	83.8	1.3	continuous	ongoing	ABC/3TC/DTG	B	
16	ID8187	Female	Black/African Descent	na	1415	na	16	74.6	33.2	2.1	continuous	ongoing	3TC/TDF/DOR	C	
17	ID8194	Male	White/Caucasian	na	363	na	26	103.0	45.9	0.6	continuous	resolved	DRV/c, DTG	B	
18	ID8198	Male	White/Caucasian	na	1286	471983	32	154.2	152.8	2.4	continuous	ongoing	FTC/TAF/BIC, MVC, DOR	B	
19	ID8208	Male	Black/African Descent	48	389	247000	3	102.0	55.3	2.2	short term ART	ongoing	FTC/TAF/BIC, DOR	B	
20	ID8216	Male	Latino/Hispanic	na	155	87411	4	171.1	85.4	3.9	short term ART	ongoing	FTC/TAF/BIC, DOR, DRV/c	B	
21	ID0468	Male	Black/African Descent	454	828	8771	9	74.1	29.9	5.2	continuous	resolved	FTC/TAF/BIC	B	P1 (15, 25)
22	ID0477	Male	White/Caucasian	197	756	na	26	87.5	70.8	14.0	continuous	ongoing	FTC/TAF/BIC, FTR	B	P2 (15, 25)
23	ID0478	Female	Black/African Descent	221	761	141667	15	174.1	178.9	8.2	continuous	ongoing	FTC/TAF/BIC	B	P3 (15, 25)
24	ID0440	Male	Black/African Descent	na	705	na	na	48.3	23.6	5.3	intermittent	ongoing	FTC/TAF/BIC	B	P5 (25)
25	ID0459	Female	Black/African Descent	536	1651	156000	23	91.8	139.6	11.0	continuous	ongoing	FTC/TAF/BIC	B	P6 (25)
26	ID0482	Male	Black/African Descent	6	273	101662	na	62.2	65.8	5.4	intermittent	resolved	FTC/TAF/RPV	B	P8 (25)
27	ID0490	Male	Latino/Hispanic	201	678	27000	4	97.8	65.4	3.8	short term ART	ongoing	CAB/RPV	B	
28	ID0491	Male	White/Caucasian	10	865	141000	27	101.9	166.7	5.2	intermittent	ongoing	FTC/TAF/BIC	B	
29	ID0492	Male	Black/African Descent	158	326	259000	na	132.5	127.8	1.2	continuous	ongoing	CAB/RPV	B	
30	ID0493	Male	White/Caucasian	4	281	4400000	3	203.4	224.0	2.0	short term ART	ongoing	FTC/TAF/BIC, DOR	B	
31	ID0572	Male	White/Caucasian	na	610	na	27	2833.8	1043.1	1.8	continuous	ongoing	FTC/TAF/BIC, DOR	B	P3 (15, 25)
32	ID0139	Male	White/Caucasian	120	1330	800000	8	40.0	25.1	2.9	intermittent	ongoing	FTC/TAF/BIC	B	ID139 (54)
			**Mean**	**203**	**737**	**404310**	**20**	**170**	**100**	**4.8**					
			standard deviation	150	382	822429	10	481	178	4					
			**Median**	**197**	**725**	**200854**	**20**	**81**	**54**	**3.5**					
			IQR	70-290	392-869	98099-478978	13-27	44-18	25-112	2-7					

Table S2. Participant characteristics – validation cohort. ART, antiretroviral therapy; NSV, nonsuppressible viremia; FTC, emtricitabine; TAF, tenofovir alafenamide; TDF, tenofovir disoproxil; BIC, bictegravir; MVC, maraviroc; DOR, doravirine; ABC, abacavir; 3TC lamivudine; DRV, darunavir; c cobicistat; EVG, elvitegravir; DTG, dolutegravir; RPV, rilpivirine; FTR, fostemsavir; CAB cabotegravir, LEN lenacapavir, LA long-acting formulation. Na indicates values not available.

#	Participant ID	Sex	Race/Ethnicity	CD4+ T cells/µL, nadir	CD4+ T cells/µL, most recent	Years on ART	HIV-1 RNA cp/mL, average during NSV	HIV-1 RNA cp/mL, standard deviation during NSV	Years of observed NSV	NSV type	Current ART	HIV-1 subtype based on u5gag	Notes
1	VC0001	Male	White/Caucasian	60	930	8.5	805	1406	8.0	continuous	CAB/RPV(LA), LEN(LA)	B	
2	VC1001	Male	White/Caucasian	na	620	20+	138	284	5.1	intermittent	DTG(bid), DOR, MVC, FTR	B	
3	VC1002	Male	White/Caucasian	210	463	14	68	35	5.1	continuous	ABC/3TC/DTG, DOR	B	
4	VC1003	Male	White/Caucasian	52	882	10+	62	41	2.8	continuous	FTC/TAF/BIC	B	
5	VC1004	Male	White/Caucasian	na	829	17	45	46	5.4	intermittent	FTC/TAF/BIC	B	
6	VC1005	Male	Hispanic/Latino	na	807	16	122	133	5.4	continuous	FTC/TAF/BIC	B	
7	VC1006	Male	White/Caucasian	198	661	13+	48	9	3.2	continuous	FTC/TAF/BIC	B	
8	VC2001	Female	Other/Hispanic	261	224	18	545	700	10.0	continuous	DRV/c, DTG(bid)	B	
9	VC2002	Male	White/Caucasian	300	787	22	269	174	12.0	continuous	FTC/TAF/BIC	B	
10	VC8348	Male	White/Caucasian	131	676	13	141	119	11.0	continuous	FTC/TAF/BIC, DOR, DRV/c, LEN(LA)	A1	
11	VC8349	Male	Asian	180	642	18	114	95	2.3	continuous	FTC/TAF/BIC	B	
12	VC8350	Male	White/Caucasian	na	1301	11	179	56	10.0	continuous	FTC/TAF, DTG, DRV/c	B	
13	VC8352	Male	White/Caucasian	570	1191	30	129	87	4.2	continuous	FTC/TAF/BIC, DOR, DRV/c	B	
14	VC0128	Male	Latino/Hispanic	22	391	2	202	93	1.4	short term ART	FTC/TAF/BIC,DRV/c	B	
15	VC0134	Male	Black/African American	133	633	26	361	124	9.0	continuous	DRV/c, DTG	B	Viral rebound due to short lapse in treatment at 6.7 years since the onset of
16	VC0135	Male	Black/African American	293	1339	15+	39	20	2.0	continuous	FTC/TAF/BIC	B	
17	VC0136	Male	Black/African American	14	605	16+	58	42	8.0	continuous	FTC/TAF/BIC	B	
18	VC0137	Male	White/Caucasian	285	739	16	100	55	9.2	continuous	FTC/TAF/BIC, DOR	B	
19	VC0479	Male	White/Caucasian	na	396	31	29	4	3.3	continuous	ABC/3TC/DTG	B	Previously published as P7 (25)
20	VC0483	Male	Black/African American	na	1016	12+	70	45	2.5	continuous	FTC/TAF/DRV/c, DTG	B	
			**Mean**	**194**	**757**	**17**	**176**	**178**	**6.0**				
			Standard deviation	142	289	8	189	319	3				
			**Median**	**189**	**708**	**17**	**118**	**71**	**5.3**				
			IQR	58-286	698-918	13-23	59-196	41-131	3-9				

Table S3. CLAWS assay oligos. The “+” sign before specific bases indicate modifications (locked nucleic acids).

#	Oligo name	Direction	5' mod	Sequence 5' to 3'	3' mod	HXB2 position	final concentration
1	claws_fwd	forward		CAGGACTCGGCTTGCTG		692-708	450nM
2	claws_msd	reverse	6-FAM	CGTAC+T+C+A+C+C	IowaBlack	743-752	125nM
3	claws_atg	reverse	HEX	CGCTCTCGCACC+C+A+T	IowaBlack	790-804	125nM
4	claws_rev	reverse		TCCCTGCTTGCCCATACTATA		888-908	450nM
