# Supplementary material for: 5′ leader defects drive persistent HIV-1 viremia on long-term ART
Source: Nat Commun. 2026 Jun 8;17:4725. doi: 10.1038/s41467-026-73475-5 (PMC13246943; doi:10.1038/s41467-026-73475-5)
Supplement: Supplementary file 2 — Reporting summary [file 41467_2026_73475_MOESM2_ESM.pdf]

nature portfolio

Corresponding author(s): NCOMMS-25-84611-B Francesco Simonetti

Last updated by author(s): May 7, 2026

Reporting Summary

Nature Portfolio wishes to improve the reproducibility of the work that we publish. This form provides structure for consistency and transparency in reporting. For further information on Nature Portfolio policies, see our Editorial Policies and the Editorial Policy Checklist.

Statistics

For all statistical analyses, confirm that the following items are present in the figure legend, table legend, main text, or Methods section.

n/a	Confirmed	
☐	☒	The exact sample size (*n*) for each experimental group/condition, given as a discrete number and unit of measurement
☐	☒	A statement on whether measurements were taken from distinct samples or whether the same sample was measured repeatedly
☐	☒	The statistical test(s) used AND whether they are one- or two-sided<br>*Only common tests should be described solely by name; describe more complex techniques in the Methods section.*
☒	☐	A description of all covariates tested
☒	☐	A description of any assumptions or corrections, such as tests of normality and adjustment for multiple comparisons
☐	☒	A full description of the statistical parameters including central tendency (e.g. means) or other basic estimates (e.g. regression coefficient) AND variation (e.g. standard deviation) or associated estimates of uncertainty (e.g. confidence intervals)
☐	☒	For null hypothesis testing, the test statistic (e.g. *F*, *t*, *r*) with confidence intervals, effect sizes, degrees of freedom and *P* value noted<br>*Give P values as exact values whenever suitable.*
☒	☐	For Bayesian analysis, information on the choice of priors and Markov chain Monte Carlo settings
☒	☐	For hierarchical and complex designs, identification of the appropriate level for tests and full reporting of outcomes
☒	☐	Estimates of effect sizes (e.g. Cohen's *d*, Pearson's *r*), indicating how they were calculated

Our web collection on statistics for biologists contains articles on many of the points above.

Software and code

Policy information about availability of computer code

Data collection	digital PCR data was collected through the software of the QIAcuity instrument (Qiagen). Sanger sequencing data of PCR products were obtained via the company Azenta. Plasmid sequences were generated via oxford nanopore sequencing by Plasmidsaurus.
Data analysis	Sanger sequencing data were analyzed via Geneious Prime software v2025.0.3. Sequence alignments were inspected via Bioedit v7.2.5. Phylogenetc analyses and trees were conducted on Mega v7.0. Statistical analyses were performed on GraphPad Prism v10.0.1. Raw dPCR data were plotted via R and the Tidyplots package. The R script for the Tidyplot package used to plot dPCR data is publicly available at: https://github.com/FRSimonetti/CLAWS_dPCR_plotting, archived at Zenodo DOI: 10.5281/zenodo.20077757

For manuscripts utilizing custom algorithms or software that are central to the research but not yet described in published literature, software must be made available to editors and reviewers. We strongly encourage code deposition in a community repository (e.g. GitHub). See the Nature Portfolio guidelines for submitting code & software for further information.

Data

Policy information about availability of data

All manuscripts must include a data availability statement. This statement should provide the following information, where applicable:

- Accession codes, unique identifiers, or web links for publicly available datasets
- A description of any restrictions on data availability
- For clinical datasets or third party data, please ensure that the statement adheres to our policy

HIV sequences are available on GenBank and the HIV database (OQ092462-OQ92467, PZ328790-PZ329638, PZ329639-PZ329991, PV927540-PV927951, PZ068438-PZ068920. There are no restrictions on the availability of these data.

Research involving human participants, their data, or biological material

Policy information about studies with human participants or human data. See also policy information about sex, gender (identity/presentation), and sexual orientation and race, ethnicity and racism.

Reporting on sex and gender	Sex and gender did not affect participant selection. This study includes a total of n = 52 individuals living with HIV-1 (Table S1). 4/52 individuals are female, 48/52 are male. The additional 10 participant with HIV from the NIH were all male. The limited representation of female individuals in our study is a direct reflection of the infected population in North America. Since most participants were enrolled from a clinic in Toronto, the population in this study reflects the HIV epidemic in Toronto, characterized mostly by men who have sex with men.
Reporting on race, ethnicity, or other socially relevant groupings	Race and ethnicity information are provided in Table S1.
Population characteristics	Relevant information such as CD4 T cell nadir, current CD4 T cell counts, ART regimens, are reported in Table S1.
Recruitment	People living with HIV on antiretroviral therapy were recruited following the inclusion criteria:<br>-Age=or>18 years<br>-On antiretroviral therapy<br>-Viral load determined by CobasTaqMan HIV-1 test v2.0 assay was detectable for multiple measurements over at least six months of observation.<br>-No evidence of poor adherence or drug resistance
Ethics oversight	All participants were adults and signed informed consent forms approved by the Institutional Review Board of the Johns Hopkins University, The University of Toronto, The University of Montreal, The university of Kansas, Rush University, And the National Institute for Allergy and Infectious Diseases.

Note that full information on the approval of the study protocol must also be provided in the manuscript.

Field-specific reporting

Please select the one below that is the best fit for your research. If you are not sure, read the appropriate sections before making your selection.

☒ Life sciences ☐ Behavioural & social sciences ☐ Ecological, evolutionary & environmental sciences

For a reference copy of the document with all sections, see nature.com/documents/nr-reporting-summary-flat.pdf

Life sciences study design

All studies must disclose on these points even when the disclosure is negative.

Sample size	We studied 32 people living with HIV with persistently or intermittently detectable viremia for at least six months. We added, in response to the reviewers' comments, a validation cohort of 20 participants with detectable viremia. A sample size calculation was not performed, since these are rare cases lacking prior knowledge of the actual frequency of 5'Leader defective RNA driving viremia.
Data exclusions	No data were excluded, with the exception of single geneome sequences with insufficient quality or because resulting from more than one HIV molecule.
Replication	Replication was tested with multiple technical replicates such as replicate culture wells (for mutant fitness experiments from Figure 3), replicate dPCR reactions, and replicate aliquots of plasma (for experiments in Figures 4-6). The correlation between CLAWS and SGS was replicated in an independent validation cohort.
Randomization	This was not a clinical trial involving randomization of participants. Moreover, randomization of experiments was not applicable to this study. Clinical samples were processed and analyzed based on when they arrived to the Simonetti Lab. No other factor determined the order of the experiments.

Blinding	This was not a clinical trial. Blinding was not necessary, as the clinical samples were not derived from participants with characteristics that would have biased the execution of the experiments or the interpretation of the data.

Reporting for specific materials, systems and methods

We require information from authors about some types of materials, experimental systems and methods used in many studies. Here, indicate whether each material, system or method listed is relevant to your study. If you are not sure if a list item applies to your research, read the appropriate section before selecting a response.

Materials & experimental systems

n/a	Involved in the study
☒	☐ Antibodies
☐	☒ Eukaryotic cell lines
☒	☐ Palaeontology and archaeology
☒	☐ Animals and other organisms
☐	☒ Clinical data
☒	☐ Dual use research of concern
☒	☐ Plants

Methods

n/a	Involved in the study
☒	☐ ChIP-seq
☒	☐ Flow cytometry
☒	☐ MRI-based neuroimaging

Eukaryotic cell lines

Policy information about cell lines and Sex and Gender in Research

Cell line source(s)	HEK293 cells were used to generate HIV in vitro. HEK293 cells were obtained from the HIV Reagents Program. https://www.beiresources.org/hiv.aspx. NR-9313
Authentication	The cell line used was not further authenticated. Transfection experiments with standard plasmid yielded the expected amounts of viral particles.
Mycoplasma contamination	Cells were negative for mycoplasma.
Commonly misidentified lines (See ICLAC register)	HEK293 cell line is not listed among commonly misidentified lines.

Clinical data

Policy information about clinical studies

All manuscripts should comply with the ICMJE guidelines for publication of clinical research and a completed CONSORT checklist must be included with all submissions.

Clinical trial registration	This study is not a clinical trial; it has no registration number
Study protocol	Given the exploratory, non interventional, non observational nature of this study, no study protocol is available. PLease refer to the Life Sciences Study Design for details regarding enrollment and inclusion criteria
Data collection	Data were collected from clinical charts and immediately de-identified, collected between 2023 and 2025
Outcomes	n/a, given its exploratory nature, this study has no primary or secondary outcome

Plants

Seed stocks	*Report on the source of all seed stocks or other plant material used. If applicable, state the seed stock centre and catalogue number. If plant specimens were collected from the field, describe the collection location, date and sampling procedures.*
Novel plant genotypes	*Describe the methods by which all novel plant genotypes were produced. This includes those generated by transgenic approaches, gene editing, chemical/radiation-based mutagenesis and hybridization. For transgenic lines, describe the transformation method, the number of independent lines analyzed and the generation upon which experiments were performed. For gene-edited lines, describe the editor used, the endogenous sequence targeted for editing, the targeting guide RNA sequence (if applicable) and how the editor was applied.*
Authentication	*Describe any authentication procedures for each seed stock used or novel genotype generated. Describe any experiments used to assess the effect of a mutation and, where applicable, how potential secondary effects (e.g. second site T-DNA insertions, mosiacism, off-target gene editing) were examined.*
