# Supplementary material for: 5′ leader defects drive persistent HIV-1 viremia on long-term ART
Source: Nat Commun. 2026 Jun 8;17:4725. doi: 10.1038/s41467-026-73475-5 (PMC13246943; doi:10.1038/s41467-026-73475-5)
Supplement: Supplementary file 3 — Transparent Peer Review file [file 41467_2026_73475_MOESM3_ESM.pdf]

nature portfolio

Peer Review File

5′ Leader Defects Drive Persistent HIV-1 Viremia on Long-Term ART

Corresponding Author: Dr Francesco Simonetti

This file contains all reviewer reports in order by version, followed by all author rebuttals in order by version.

Version 1:

Reviewer comments:

Reviewer #1

(Remarks to the Author)
The authors' prior work has offered important insights into a potential mechanism underlying the clinical phenomenon of "nonsuppressible viremia," demonstrating that defects within the HIV-1 5′-leader region can give rise to replication-incompetent virions. This observation is clinically meaningful, as it helps prevent unnecessary regimen changes by showing that such low-level viremia does not indicate emerging drug resistance but instead highlights an underappreciated pitfall: detectable plasma HIV-1 RNA does not always correspond to active, replication-competent virus. In the present study, the authors introduce a novel dPCR assay designed to detect sequence defects (both substitutions and small deletions) within the HIV-1 5′-leader region by employing a drop-off probe strategy. This methodological approach enables rapid screening for 5′-leader abnormalities without the need for direct sequencing and represents a natural and logical extension of the authors' prior work elucidating the virological basis of nonsuppressible viremia.

Major concerns:
1) The central contribution of this study is the introduction of a novel duplex dPCR drop off assay, called CLAWS (Capturing 5' Leader Anomalies Without Sequencing). However, the specific probe configuration used in the present study warrants careful reconsideration in light of the technical limitations outlined below. In the duplex dPCR drop-off design, the absence of FAM signal constitutes the analytical "event," which is interpreted as indicative of droplets containing putative deletions and/or substitutions within the MSD region in the CLAWS assay. However, any technical loss of FAM signal (including low probe Tm, suboptimal annealing temperature, probe degradation, or day-to-day variation) is indistinguishable from a true biological drop-off event. The MSD probe is intrinsically disadvantaged by suboptimal hybridization thermodynamics: its estimated Tm is 57 °C (as calculated using IDT OligoAnalyzer and Qiagen's Tm Calculator), whereas the assay is performed at 61 °C (Page 29, line 614). In contrast, the HEX-labeled ATG probe has a substantially higher Tm of 68 °C, supporting more efficient and stable annealing. Because the assay interprets loss of FAM signal as evidence of either deletion of the corresponding nucleotide stretch or substantial nucleotide mismatches in the 5′-leader region, the use of a thermodynamically suboptimal drop-off probe increases the likelihood that suboptimal hybridization, arising from technical factors rather than genuine sequence defects, will generate FAM-negative droplets and thereby inflate the apparent frequency of MSD abnormalities. In addition, the 10-nt design of the FAM-labeled MSD probe is intrinsically suboptimal for use as a drop-off probe. At this length, the fluorophore and quencher are in close physical proximity, such that even after probe cleavage the resulting fragments may remain sufficiently near one another to permit continued quenching. Although locked nucleic acids (LNAs) were incorporated to elevate the probe's melting temperature, the combination of LNA bases and the inherently short 10-nt architecture renders the probe susceptible to over-quenching and reduced fluorescence amplitude. Consistent with this, issues related to depressed FAM signal intensity are evident in Figures 4D, 5A, 5B, and 6D, where the FAM amplitude consistently reaches only approximately half that of the HEX-labeled ATG probe. This disparity is particularly striking in Figure 6D, where the FAM signal is markedly reduced (approximately 25) relative to the HEX signal (approximately 90). Collectively, this diminished reporter amplitude substantially compromises the probe's effectiveness as a reliable drop-off indicator.

2) Because this manuscript aims to introduce a novel assay, the authors have undertaken extensive efforts to validate its performance using cohorts drawn from multiple clinical studies. While detection of the targeted phenomenon with the new assay may be interpreted as evidence of assay success, such conclusions should be made with caution. If the cohorts selected for evaluation are enriched for the very features the assay was designed to detect, the observed performance may primarily reflect sampling bias rather than true assay robustness. In these circumstances, additional evaluation in more

diverse or unselected populations is necessary to determine whether the assay can generalize beyond the initial, highly tailored cohort. It is important to establish that the assay can detect this phenotype de novo in an unselected population, rather than only within cohorts pre-enriched for the target characteristics.

3) The data presented in Figures 1-3 largely reiterate clinical observations that have already been well documented in the literature (including their own previous studies) and, as currently displayed, do not meaningfully advance the central message of the manuscript. Among these datasets, the most substantive new contribution is the detailed characterization of the precise locations and sequence patterns of defects, including short nucleotide deletions within the 5′-leader region, as shown in Figure 3. However, the current presentation does not sufficiently highlight this important finding. Instead, the figure set gives the impression that similar analyses were repeated across different cohorts without yielding additional insight. The authors may wish to streamline the figures or consolidate overlapping panels into a more concise format to improve clarity and better emphasize the novel aspects of the study.

4) The manuscript presents an extensive set of validation and exploratory analyses derived from a relatively limited dataset (Figures 4-6), resulting in a large number of visualizations that ultimately obscure a clear and cohesive message. Although these efforts reflect considerable analytical work, the breadth of figures and exploratory plots makes it difficult to identify the primary conclusions supported by the data. Moreover, many of these results are not clearly integrated into the narrative and do not effectively highlight the unique capabilities of the assay. I recommend prioritizing the most rigorous and informative analyses, consolidating or removing secondary exploratory figures, and more clearly articulating the key findings that the dataset can robustly support.

Minor concerns and suggestions:
5) The authors briefly address the definition or criteria for nonsuppressible viremia (NSV), describing it as detectable plasma HIV-1 RNA (>20–50 copies/mL) in individuals on ART (page 3, lines 67-68), potentially including those in whom optimization or intensification of therapy has failed to reduce viremia (page 4, lines 94-95). For clarity, it would be helpful for the authors to specify whether a commonly accepted definition or set of diagnostic criteria for NSV exists in the field. If no consensus definition is established in the literature, the authors should explicitly articulate the operational definition of NSV used in this study. In addition, specifying the duration of viremia required to classify an individual as having NSV, whether this follows accepted conventions or reflects the authors' own criteria, would further improve interpretability.

6) The authors should discuss the potential molecular mechanisms underlying the loss of short nucleotide stretches in the 5′-leader region. Notably, based on the observed boundary sequence motifs, these deletions do not appear to be attributable to canonical RNA splicing events. In addition, the authors should consider and address the possibility that the observed 5′-leader deletions might arise as artifacts during cDNA synthesis or PCR amplification rather than representing biologically relevant events.

7) The duplex dPCR assay developed in this study employs a FAM-labeled MSD drop-off probe and a HEX-labeled ATG reference probe to detect HIV-1 RNA species that harbor defects in the 5′-leader region while retaining the gag start codon, presumably indicating preservation of an intact Gag open reading frame, which is essential for virus particle production. It would be helpful for the authors to comment on whether 5′-leader defects are expected to occur exclusively in the context of genomes that remain gag-intact. Alternatively, is it plausible that 5′-leader defects may also arise in transcripts derived from proviruses with larger internal deletions extending into downstream regions of gag (e.g., loss of the 3′ end of gag), as is commonly observed in defective proviruses? Clarification on this point would assist readers in interpreting the biological relevance and diagnostic implications of the assay's readouts.

8) On page 3, lines 66-67, the author states "Trace plasma HIV-1 RNA, or residual viremia (RV), is detected in more than half of PWH on ART." The phrasing "Trace plasma…" appears to contain a typographical or grammatical error. A clearer and more precise formulation would be: "Trace levels of plasma HIV-1 RNA, also referred to as residual viremia (RV), are detected in more than half of PWH on ART."

9) On page 3, lines 70-71, the sentence "NSV, raises concerns for virologic failure, transmission, and immune activation, complicates ART management, causing anxiety and ultimately affecting the quality of life of PWH" reads as overly compressed and contains multiple concepts strung together without clear logical transitions. I recommend revising this statement to improve clarity and flow, for example, by separating distinct clinical and psychosocial implications into discrete, well-structured clauses.

10) On page 3, lines 72-74, "Its prevalence seems to be rising, likely reflecting the higher sensitivity of clinical assays and the growing population on long-term ART." When a phenomenon appears to be increasing in prevalence, it is important to consider that this trend may reflect enhanced detection rather than a true rise. As more individuals actively look for the phenomenon through increased testing, surveillance, or reporting, the likelihood of identifying and documenting cases naturally increases, potentially creating the impression of a growing prevalence even if the underlying incidence has not changed.

11) On page 5, line 105, and page 26, line 544, the authors refer to the HIV-1-specific primer used for cDNA synthesis. For transparency and reproducibility, the nucleotide sequence of this primer should be provided, at minimum within the Methods section (page 26, line 544).

12) On pages 18-19 and in Figure 6, the authors assert that the NSV variant sequences remained unchanged (i.e., exhibited no measurable evolution) over the 3-year observational period; that viruses rebounding during analytical treatment

interruption (ATI) were largely distinct from the NSV variants and therefore that the sources giving rise to NSV prior to ATI were unlikely to represent the source of viral rebound; and that newly seeded viral reservoirs following ATI may subsequently contribute to NSV. Although these interpretations are plausible and conceptually sound, the manner in which the data are presented does not adequately support or clearly convey these conclusions. Moreover, the analysis appears to rely on a single illustrative case, potentially rendering the findings anecdotal rather than broadly generalizable.

13) On page 26, lines 552 and 556, the authors report using VeriFi Polymerase (PCR Biosystems) for the outer PCR and Platinum Taq High Fidelity DNA Polymerase (Thermo Fisher Scientific) for the nested PCR. The rationale for employing different polymerases at these two amplification steps should be clarified.

Reviewer #2

(Remarks to the Author)
In a previous study (White et al., 2023), the authors identified MSD-defective proviruses (termed 5′L-defective here) as a source of non-suppressible viremia (NSV) in plasma of ART-treated participants. Although underrepresented in the overall proviral landscape, highly clonal MSD-defective viruses were shown to be the predominant drivers of NSV. The authors demonstrated that MSD-defective proviruses were not replication-competent but retained partial viral gene expression and genomic RNA packaging capacity. Importantly, defective proviruses causing NSV evaded cellular immune pressure by displaying CTL escape mutations and were not neutralized by autologous IgGs. When expressed, these defective proviruses can be recognized by the host immune system, potentially distracting CTL responses from eliminating the latent reservoir and contributing to chronic immune activation and inflammation during ART.
In the present study, Box et al. extend their previous observations to a larger cohort of participants exhibiting NSV despite effective ART. Using sequencing of the 5′L-gag and p6/protease/RT regions, the authors confirmed prior findings and added new data from pre-analytical treatment interruption (ATI), rebound, and post-ATI plasma samples. These results demonstrated that 5′L-defective viruses predominated in plasma at all time points, except during rebound when 5′L-intact viruses became predominant. To enable rapid profiling of 5′L RNA intactness, the authors developed and validated the CLAWS dPCR assay to quantify intact and defective 5′L viruses. Comparison of sequencing and dPCR results showed that CLAWS is a practical tool for clinical assessment of persistent viremia and HIV-1 cure trials.
The broad range of experimental settings used to validate the CLAWS dPCR assay is convincing and makes this paper highly relevant by presenting a new tool to explore the dynamics of residual viremia and to assess curative interventions.
Comments
1# In addition to the "claws-msd" probe and "claws-rev" primer, the CLAWS dPCR assay was designed using nearly identical primers/probes as those used in IPDA. To gain further insights into the "true" intactness of plasma viruses by assessing the presence of both 5′L and Env regions, did the authors attempt to combine CLAWS primers/probes with IPDA Env primers/probes in a multiplex format?
Although RNA processing and degradation could induce shearing that might limit the detection of full-length genomes, combining CLAWS and IPDA assays (or at least testing for correlation between 5′L and Env intactness) might have provided more informative data on plasma virus and provirus intactness. This would have strengthened the study by clarifying whether 5′L-intact viruses in plasma also retain intact Env regions, which is critical for assessing replication competence.

2# As a corollary to Comment 1, do the authors have any evidence regarding the actual genome-wide intactness of 5′L-intact viruses detected in plasma? While the manuscript demonstrates that 5′L-defective sequences dominate NSV, it remains unclear whether the minority population of 5′L-intact sequences represents fully replication-competent virus or harbors defects in other genomic regions (e.g., env, accessory genes).
This information is critical for refining cure strategies, as it would clarify whether: 1) 5′L-intact viruses in plasma during NSV represent a persistent source of replication-competent virus. 2) These viruses could contribute to viral rebound upon ART interruption. 3) Therapeutic interventions should differentially target 5′L-intact versus 5′L-defective reservoirs

3# While 5′L-defective sequences dominate HIV-1 RNA in the plasma of ART-treated participants, 5′L-intact sequences were detected in 18 of 31 participants (58%) (Figure 2A). Given that these 5′L-intact sequences, though representing a minority of total NSV, were present in the majority of participants, how can the authors rule out the possibility that this small fraction of NSV contributes meaningfully to viral persistence in ART-treated participants?
*Could these 5′L-intact viruses represent low-level replication-competent virus production?
*Do participants with detectable 5′L-intact NSV show different clinical outcomes (e.g., time to rebound after ATI, reservoir size, immune activation markers)?
*Should this population be considered clinically irrelevant to HIV-1 pathogenesis, or does it warrant closer monitoring?
In conclusion: This is a well-executed study that makes significant contributions to our understanding of HIV persistence and provides a valuable new tool (CLAWS) for the field. The major comments raised above, if addressed, would further strengthen the manuscript's impact and clinical utility.

Reviewer #3

(Remarks to the Author)
Box et al present sequence analysis of plasma HIV sequence in 32 PWH who have non-suppressible viremia (NSV), showing that a large proportion of plasma HIV RNA in this population contains 5-Leader defects that cluster around the MSD, results that expand earlier findings from the same group and others. They go on to develop a dPCR assay to quickly and easily discern MSD defects in plasma. The paper is overall well-written, the methodology sound, will be of high interest to the HIV remission field and may also have important clinical implications. Some clarification throughout the text will be

helpful, particularly regarding how proportions of clonal variants relate to the overall finding that 95% of plasma viremia consists of defective variants.

Major comments:
-The description in the abstract of the proportions of variants is unclear (lines 39-41). Adding the breakdown of clonal variants that contributes to the 95% of defective plasma variants may help lead into the next sentence and clarify how the variant proportions contribute to the overall amount of defectives.
Minor comments:
-In Figure 1A authors should label the red dotted line.
-Line 143: Please define “regions of length polymorphism”, or use a different term.
-Line 186: As this is the first mention of the 91 unique variants (besides the abstract), it should be defined/included earlier in the manuscript, perhaps in the first section where variant amounts and proportions are discussed (around lines 109-110, and/or line 140 perhaps?).
-Figure 3C: The labels for the White and Mohammadi papers appear to go with 3D. Consider changing the placement.
-Figure 3E figure legend: Participant is labeled as ‘AU0139’ rather than ‘ID0139’.
-Lines 273-274 and Fig 3: This would be better visualized if the different deletions (including delta97) were labeled on Figure 3 next to their corresponding sequences. This way reader can better visualize the deletions in relation to primer binding sites.
-Figures 4 and S7: Clarity would be enhanced by a schematic showing the genome with primer/probe binding sites for each assay.
-Section starting on line 332: In addition to the comment above some additional context about what the IPDA measures vs what the CLAWS assay measures and what similarities/differences in results would be expected based on current knowledge (for example, IPDA interrogates a 3’ region as well as 5’, how would results be interpreted given CLAWS does not have a 3’ counterpart).
-Figure 5B: It would be helpful if the % of intact and defective 5’L sequences could be added on the left side (the sequencing results) to be more easily compared with the % that is given on the right for the CLAWS data.
-Line 395-396: Is there data from a different participant that can be used? This participant’s plasma viral RNA sequences contained a mutation that abolished detection with the CLAWS assay, necessitating participant-specific assay design. It would be nice to see data that illustrates the CLAWS assay working as designed on samples from a clinical trial. Of course, clinical samples are limited and this is not always possible. Just something to consider.
-Line 406: There is no call-out for Fig 6F.
-Line 428: Do the authors mean “defective viral RNA” rather than “defective proviruses”?
-Line 447: I suggest authors re-word the sentence starting with “Thus, these proviruses may be released from…” to enhance clarity. For example, the word “released” is a bit confusing. Perhaps something like “these proviruses may evade CPE and immune clearance, leading to positive selection over time”
-Line 452-454: Do other proviruses retain expression of Nef? If so, 5’L defective proviruses would not necessarily have an advantage over others.
-Line 464-466: Authors should add “always or usually” and “could be”, since there is still a possibility that detectable viral load is from replication (in cases of drug resistance). “First, periods of detectable viral load in adherent PWH should not always/usually be interpreted as evidence of replication, but rather could be virus released from defective proviruses”

Version 2:

Reviewer comments:

Reviewer #1

(Remarks to the Author)
I thank the authors for their careful revision of the manuscript and for the additional analyses provided. The authors have adequately addressed the concerns raised by the reviewers, and the revisions have improved the clarity and rigor of the study. In my opinion, the manuscript is now suitable for publication.

Reviewer #2

(Remarks to the Author)
This reviewer is satisfied by the revised manuscript.

Reviewer #3

(Remarks to the Author)
The authors have sufficiently addressed our initial comments in their revision.

Open Access This Peer Review File is licensed under a Creative Commons Attribution 4.0 International License, which permits use, sharing, adaptation, distribution and reproduction in any medium or format, as long as you give appropriate credit to the original author(s) and the source, provide a link to the Creative Commons license, and indicate if changes were made.
In cases where reviewers are anonymous, credit should be given to 'Anonymous Referee' and the source.
The images or other third party material in this Peer Review File are included in the article's Creative Commons license, unless indicated otherwise in a credit line to the material. If material is not included in the article's Creative Commons license and your intended use is not permitted by statutory regulation or exceeds the permitted use, you will need to obtain permission directly from the copyright holder.
To view a copy of this license, visit https://creativecommons.org/licenses/by/4.0/

REVIEWER COMMENTS

We thank the reviewers for their helpful comments and suggestions. Our point-by-point responses are highlighted in *blue*. Key changes in the manuscript text are highlighted in **green**.

Reviewer #1 (Remarks to the Author):

The authors' prior work has offered important insights into a potential mechanism underlying the clinical phenomenon of "nonsuppressible viremia," demonstrating that defects within the HIV-1 5′-leader region can give rise to replication-incompetent virions. This observation is clinically meaningful, as it helps prevent unnecessary regimen changes by showing that such low-level viremia does not indicate emerging drug resistance but instead highlights an underappreciated pitfall: detectable plasma HIV-1 RNA does not always correspond to active, replication-competent virus. In the present study, the authors introduce a novel dPCR assay designed to detect sequence defects (both substitutions and small deletions) within the HIV-1 5′-leader region by employing a drop-off probe strategy. This methodological approach enables rapid screening for 5′-leader abnormalities without the need for direct sequencing and represents a natural and logical extension of the authors' prior work elucidating the virological basis of nonsuppressible viremia.

We thank Reviewer #1 for their constructive review and for highlighting both the clinical significance of 5'Leader defects in nonsuppressible viremia and the value of the CLAWS dPCR approach. All concerns have been addressed below and in the revised manuscript.

Major concerns:

1) The central contribution of this study is the introduction of a novel duplex dPCR drop off assay, called CLAWS (Capturing 5' Leader Anomalies Without Sequencing). However, the specific probe configuration used in the present study warrants careful reconsideration in light of the technical limitations outlined below. In the duplex dPCR drop-off design, the absence of FAM signal constitutes the analytical "event," which is interpreted as indicative of droplets containing putative deletions and/or substitutions within the MSD region in the CLAWS assay. However, any technical loss of FAM signal (including low probe Tm, suboptimal annealing temperature, probe degradation, or day-to-day variation) is indistinguishable from a true biological drop-off event. The MSD probe is intrinsically disadvantaged by suboptimal hybridization thermodynamics: its estimated Tm is 57 °C (as calculated using IDT OligoAnalyzer and Qiagen's Tm Calculator), whereas the assay is performed at 61 °C (Page 29, line 614). In contrast, the HEX-labeled ATG probe has a substantially higher Tm of 68 °C, supporting more efficient and stable annealing. Because the assay interprets loss of FAM signal as evidence of either deletion of the corresponding nucleotide stretch or substantial nucleotide mismatches in the 5′-leader region, the use of a thermodynamically suboptimal drop-off probe increases the likelihood that suboptimal hybridization, arising from technical factors rather than genuine sequence defects, will generate FAM-negative droplets and thereby inflate the apparent frequency of MSD abnormalities. In addition, the 10-nt design of the FAM-labeled MSD probe is intrinsically suboptimal for use as a drop-off probe. At this length, the fluorophore and quencher are in close physical proximity, such that even after probe cleavage the resulting fragments may remain sufficiently near one another to permit continued quenching. Although locked nucleic acids (LNAs) were incorporated to elevate the probe's melting temperature, the combination of LNA bases and the inherently short 10-nt architecture renders the probe susceptible to over-quenching and reduced fluorescence amplitude. Consistent with this, issues related to depressed FAM signal intensity are evident in Figures 4D, 5A, 5B, and 6D, where the FAM amplitude consistently reaches only approximately half that of the HEX-labeled ATG probe. This disparity is particularly striking in Figure 6D, where the FAM signal is markedly reduced (approximately 25) relative to the HEX signal (approximately 90). Collectively, this diminished reporter amplitude substantially compromises the probe's effectiveness as a reliable drop-off indicator.

1) We thank the reviewer for their careful and technically sophisticated evaluation of the probe design. We agree that the MSD (FAM-labeled) probe exhibits atypical features, including a relatively low melting temperature,

reduced mean fluorescence amplitude of positive events, and a shorter-than-usual probe length. While these characteristics could reasonably be interpreted as limitations in a conventional drop-off assay, they were deliberately selected to enable CLAWS to achieve a challenging analytical objective: the sensitive detection of HIV-1 RNA while ensuring complete signal loss in the presence of even minimal sequence disruptions within the MSD region, including single-nucleotide substitutions or very small deletions.

In this context, a more thermodynamically robust probe would indeed be expected to generate stronger fluorescence; however, this would come at the expense of specificity, as such a probe would be more tolerant of mismatches and therefore less capable of discriminating against the diverse and heterogeneous defects observed in vivo. CLAWS was not designed to exclude a single predefined mutation, but rather to capture a broad spectrum of biologically relevant 5'Leader defects. Consistent with this goal, the sequencing of samples from 31 individuals with nonsuppressible viremia revealed 31 distinct defect patterns, necessitating an assay design that prioritizes mismatch discrimination. The MSD probe configuration was therefore empirically optimized to balance sensitivity and specificity in the context of this biological diversity.

The reviewer also raises an important concern regarding the short probe length and the potential for over-quenching to reduce fluorescence amplitude. While a longer probe would, in principle, be preferable, probe length in this region is constrained by the extraordinary genetic diversity of HIV-1. Our prior experience with the IPDA assay, which employs a longer probe targeting the same 5'Leader region (now illustrated in revised Fig. S7A), demonstrated frequent amplification failures due to naturally occurring polymorphisms at probe termini. Notably, the nucleotide at position 742 is highly polymorphic, with approximately 10% of people with HIV-1 carrying a T-to-C substitution, necessitating its exclusion from the CLAWS design. Additional nucleotides immediately 3' of the MSD are also highly variable and represent a well-documented source of IPDA failure (PMID: 33986282). These constraints limited the probe to the most conserved 10-nucleotide stretch, five of which fall within the MSD and are disproportionately affected by 5'Leader defects. Importantly, because Taq polymerase cleaves probes nucleotide by nucleotide via its 5' exonuclease activity, fluorophore release occurs immediately upon probe hydrolysis, independent of the fluorophore–quencher distance.

Finally, the reviewer is concerned that technical loss of FAM signal could be indistinguishable from bona fide MSD defects. While this is theoretically possible, we addressed this risk through extensive analytical validation. CLAWS was evaluated using i) plasma pooled from multiple donors and treated with the same anti-coagulant as our clinical samples, ii) synthetic gene blocks, iii) virus generated in vitro, and iv) standardized HIV RNA reference materials from the Virus Quantification Assurance program at Duke University. Across these systems, CLAWS demonstrated high sensitivity and strong agreement with sequencing-based measurements, with only a minimal bias (2.4%) in estimating the proportion of 5'Leader–defective RNA. As suggested by the reviewer in the subsequent comment, we further extended this comparison to an independent validation cohort (see response to comment #2). CLAWS's strong agreement with sequence analysis was confirmed in this validation cohort (Fig. 5). Importantly, the reduced FAM amplitude noted by the reviewer does not translate into reduced analytical performance. In Figure 6, where patient-specific probe customization was implemented to further mitigate a patient-specific sequence polymorphism, CLAWS measurements closely matched single-genome sequencing across samples. This concordance demonstrates that, despite lower absolute FAM fluorescence intensity, droplet classification remains robust and the assay accurately quantifies 5'Leader defects at the individual-patient level.

2) Because this manuscript aims to introduce a novel assay, the authors have undertaken extensive efforts to validate its performance using cohorts drawn from multiple clinical studies. While detection of the targeted phenomenon with the new assay may be interpreted as evidence of assay success, such conclusions should be made with caution. If the cohorts selected for evaluation are enriched for the very features the assay was designed to detect, the observed performance may primarily reflect sampling bias rather than true assay robustness. In these circumstances, additional evaluation in more diverse or unselected populations is necessary to determine whether the assay can generalize beyond the initial, highly tailored cohort. It is important to establish

that the assay can detect this phenotype de novo in an unselected population, rather than only within cohorts pre-enriched for the target characteristics.

2) We thank the reviewer for raising this important point regarding potential sampling bias and assay generalizability. To directly address this concern, we evaluated CLAWS in an independent validation cohort of 20 newly enrolled participants who were not included in prior 5'Leader analyses. These individuals were referred to us by HIV-1 specialists because of persistent or intermittent detectable plasma HIV-1 RNA in the preceding months and were not pre-characterized for 5'Leader defects or other clinical or viro-immunological features. Participant characteristics are summarized in Table S2.

In this validation cohort, we quantified the proportion of 5'Leader–intact plasma HIV-1 RNA using both 5'L-gag single-genome sequencing (generating 482 new sequences) and CLAWS, as we did for the original cohort. The median percentage of 5'Leader–intact RNA was 2.5 (IQR, 0–82) by single-genome sequencing and 9.7 (IQR, 0.8–85.8) by CLAWS (p=0.4), consistent with the original cohort (Fig S6C). The two assays showed strong concordance (Spearman r=0.92, p<0.0001, see figure below). These results are presented in Fig. 5, in the new supplemental figure S6, and are described in the revised Results section. In Fig. 5G, we updated the bias analysis (Bland-Altman plot) between single genome sequencing and CLAWS, showing only a mean bias of -1.9% (95%CI, -18,+15%).

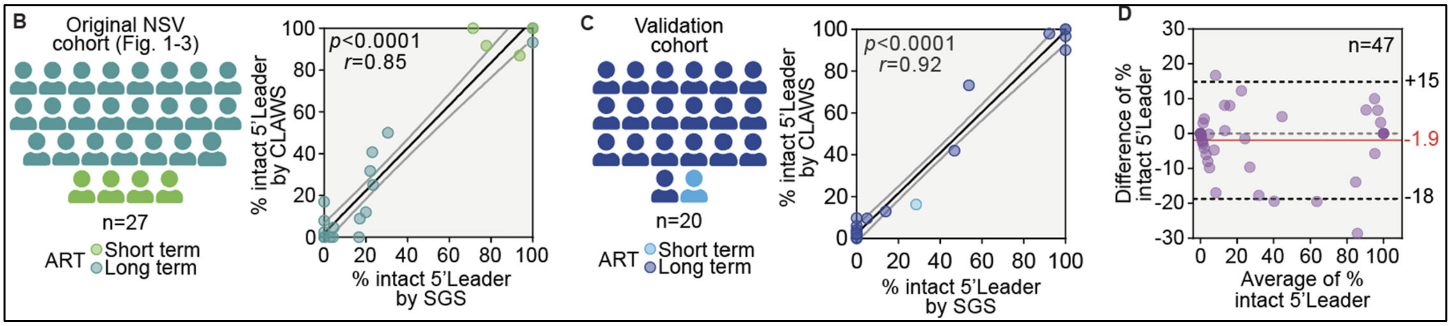

Together, these data demonstrate that CLAWS reliably distinguishes intact from defective MSD RNA in an independent multi-center cohort, supporting its robustness beyond the initial study population. Moreover, the addition of the validation cohort allowed us to obtain 5'Leader sequencing RNA data from a total of 51 PWH with NSV, further strengthening our findings that virus populations with MSD defects dominate plasma HIV-1 RNA on suppressive ART.

3) The data presented in Figures 1-3 largely reiterate clinical observations that have already been well documented in the literature (including their own previous studies) and, as currently displayed, do not meaningfully advance the central message of the manuscript. Among these datasets, the most substantive new contribution is the detailed characterization of the precise locations and sequence patterns of defects, including short nucleotide deletions within the 5′-leader region, as shown in Figure 3. However, the current presentation does not sufficiently highlight this important finding. Instead, the figure set gives the impression that similar analyses were repeated across different cohorts without yielding additional insight. The authors may wish to streamline the figures or consolidate overlapping panels into a more concise format to improve clarity and better emphasize the novel aspects of the study.

3) We thank the reviewer for this constructive comment. We agree that the most novel biological insight in the study is the fine-scale mapping of 5' Leader defects, including single point mutations, as shown in Fig. 3. In the revised manuscript, we have streamlined Figures 1–3 to more clearly emphasize this contribution and to reduce redundancy.

We note, however, that the clinical datasets in Figs. 1–2 serve an important role beyond reiterating prior observations, especially for the audience of clinicians. Previous studies of nonsuppressible viremia have been limited to small cohorts (n= 4–8), whereas our analysis of 32 individuals (plus the new 20 individuals from the

validation cohort) represents the largest clinical characterization of NSV to date. Currently, among the major HIV guidelines, only the European AIDS Clinical Society guidelines mention NSV from cellular proliferation in fully adherent patients without drug resistance as a potential reason for persistent viraemia. Thus, reaching a consensus on what constitutes possible NSV is a priority, both to raise awareness among clinicians and to provide standard criteria when designing clinical studies of NSV. Given the increasing recognition of NSV and the limited guidance in current clinical guidelines, these data provide a necessary framework for interpreting molecular analyses and for better defining the clinical context of individuals presenting with NSV (challenges reviewed in Esteban-Cantos et al., PMID: 38604202).

To better align the figure set with the central message of the manuscript, we have consolidated overlapping panels. These changes improve clarity and more directly foreground the novel aspects of the study.

4) The manuscript presents an extensive set of validation and exploratory analyses derived from a relatively limited dataset (Figures 4-6), resulting in a large number of visualizations that ultimately obscure a clear and cohesive message. Although these efforts reflect considerable analytical work, the breadth of figures and exploratory plots makes it difficult to identify the primary conclusions supported by the data. Moreover, many of these results are not clearly integrated into the narrative and do not effectively highlight the unique capabilities of the assay. I recommend prioritizing the most rigorous and informative analyses, consolidating or removing secondary exploratory figures, and more clearly articulating the key findings that the dataset can robustly support.

4) We thank the reviewer for this thoughtful and constructive feedback. We agree that the original presentation of Figures 4–6 placed emphasis on analytical breadth at the expense of a clearly articulated central message.

In response, we have streamlined this section of the manuscript to prioritize the most rigorous and informative analyses that directly support the primary conclusions. The more exploratory panels have been consolidated or moved to the supplementary figures, and the remaining figures have been reorganized to focus on the unique capability of CLAWS to quantify 5' Leader–intact versus defective plasma HIV RNA.

Minor concerns and suggestions:

5) The authors briefly address the definition or criteria for nonsuppressible viremia (NSV), describing it as detectable plasma HIV-1 RNA (>20–50 copies/mL) in individuals on ART (page 3, lines 67-68), potentially including those in whom optimization or intensification of therapy has failed to reduce viremia (page 4, lines 94-95). For clarity, it would be helpful for the authors to specify whether a commonly accepted definition or set of diagnostic criteria for NSV exists in the field. If no consensus definition is established in the literature, the authors should explicitly articulate the operational definition of NSV used in this study. In addition, specifying the duration of viremia required to classify an individual as having NSV, whether this follows accepted conventions or reflects the authors' own criteria, would further improve interpretability.

5) We thank the reviewer for highlighting the need for greater clarity regarding the definition of nonsuppressible viremia (NSV). At present, there is no universally accepted or formally standardized definition of NSV. Rather, the term has been used descriptively to refer to low-level plasma HIV-1 RNA despite ART adherence and the absence of detectable drug resistance, with variability across studies in viral load thresholds and duration criteria (PMID: 38604202).

Guidelines for antiretroviral treatment of HIV from the International AIDS Society–USA Panel and the US Department of Health and Human Service define LLV (low-level viremia) as HIV RNA levels between 50 and 200 copies per mL, the WHO recommendations increase the upper limit to 1000 copies per mL, and the European AIDS Clinical Society guidelines do not provide a specific definition.

To address this, we have revised the manuscript to explicitly state the operational definition of NSV used in this study. Specifically, we defined NSV as persistent or intermittent detection of plasma HIV-1 RNA above 20

copies/mL in individuals on ART for at least 6 months, despite documented adherence and without evidence of virologic failure attributable to drug resistance or suboptimal therapy. The duration threshold used (six months) reflects prior reports and clinical practice in which transient viral “blips” are distinguished from sustained viremia.

We have clarified the lack of a standardized definition of NSV in the Introduction, and clearly stated our operational definition of NSV at the beginning of the results section.

6) The authors should discuss the potential molecular mechanisms underlying the loss of short nucleotide stretches in the 5′-leader region. Notably, based on the observed boundary sequence motifs, these deletions do not appear to be attributable to canonical RNA splicing events. In addition, the authors should consider and address the possibility that the observed 5′-leader deletions might arise as artifacts during cDNA synthesis or PCR amplification rather than representing biologically relevant events.

6) We thank the reviewer for this helpful suggestion. Based on the sequence boundaries of the observed deletions, we agree that these events are not consistent with RNA splicing. Instead, available evidence supports their origin during reverse transcription, likely during late stages of minus-strand cDNA synthesis. As previously described (PMID: 36602866), short deletions can be favored through microhomology-mediated template switching by HIV reverse transcriptase, a process that occurs repeatedly during cDNA synthesis (PMID: 19721086). This mechanism is consistent with recurring defects such as the d22 deletion, which is flanked by short homologous “GAG” repeats (PMID: 36602866). Moreover, homopolymeric regions (such as the stretch of As and Ts downstream of the MSD, part of the encapsidation core signal region) can promote RT stalling and consequent short insertions/deletions.

Although the likelihood of generating proviruses with these defects is low, they can be observed in single-round infection experiments (PMID: 27500724). Albeit rare, 5’Leader defective proviruses accumulate during untreated infection and are further positively selected when viral replication is blocked by ART, because they are significantly less detrimental for cell survival than intact proviruses (the same phenomenon happens with proviruses that are defective because of G-to-A hypermutation caused by APOBEC3G/F). In agreement with previous work from our group and others (PMID: 36602866, 40802737, 37957382), in this study we directly identified proviruses in CD4+ T cell-derived DNA harboring corresponding 5'L defects that serve as templates for defective plasma RNA (Fig. 2E, 2G), supporting a biological rather than artifactual origin.

We also considered the possibility that these deletions could arise during cDNA synthesis or PCR amplification. To minimize such artifacts, RNA was denatured prior to reverse transcription and cDNA synthesis was performed at higher temperatures to reduce secondary structure. Moreover, the recurrent recovery of identical 5' Leader deletions in a participant-specific manner, across independent molecules, argues strongly against stochastic in vitro artifacts and supports their in vivo origin.

We have briefly addressed these points in the introduction.

7) The duplex dPCR assay developed in this study employs a FAM-labeled MSD drop-off probe and a HEX-labeled ATG reference probe to detect HIV-1 RNA species that harbor defects in the 5′-leader region while retaining the gag start codon, presumably indicating preservation of an intact Gag open reading frame, which is essential for virus particle production. It would be helpful for the authors to comment on whether 5′-leader defects are expected to occur exclusively in the context of genomes that remain gag-intact. Alternatively, is it plausible that 5′-leader defects may also arise in transcripts derived from proviruses with larger internal deletions extending into downstream regions of gag (e.g., loss of the 3′ end of gag), as is commonly observed in defective proviruses? Clarification on this point would assist readers in interpreting the biological relevance and diagnostic implications of the assay’s readouts.

7) We thank the reviewer for this thoughtful question. Importantly, the 5' Leader defects detected by CLAWS in plasma RNA do not originate at the transcript level but instead arise during the generation of proviruses, during reverse transcription, and are subsequently transcribed from these defective proviral templates.

In plasma, the striking predominance of 5' Leader–defective RNA genomes that retain an intact gag ORF reflects a biological constraint rather than an exclusive mode of defect formation. Viral assembly and particle formation, RNA packaging, and release of virions into plasma require functional Gag; consequently, proviruses harboring larger deletions that disrupt gag are unlikely to contribute measurably to circulating virions, even if transcriptionally active. As a result, plasma HIV-1 RNA is strongly enriched for genomes that preserve gag gene integrity, while defective proviruses with extensive gag deletions are largely invisible at the level of virion-associated RNA.

In contrast, proviral sequencing studies independent of virus production (such as PMID: 24243014, 27500724, 29045846) have shown that most defective proviruses contain large internal deletions often affecting gag, while a smaller fraction shows shorter deletions involving the PSI/MSD region. In some cases, these deletions extend into the gag ORF. Notably, this type of analysis of 5'Leader versus Gag intactness has not been formally addressed, and should be investigated in future studies. However, the highest density of missing nucleotides is consistently observed around the MSD (PMID: 30700913), consistent with the defects captured by CLAWS.

8) On page 3, lines 66-67, the author states "Trace plasma HIV-1 RNA, or residual viremia (RV), is detected in more than half of PWH on ART." The phrasing "Trace plasma…" appears to contain a typographical or grammatical error. A clearer and more precise formulation would be: "Trace levels of plasma HIV-1 RNA, also referred to as residual viremia (RV), are detected in more than half of PWH on ART."

8) We thank the reviewer for their suggestion. We modified the text as suggested.

9) On page 3, lines 70-71, the sentence "NSV, raises concerns for virologic failure, transmission, and immune activation, complicates ART management, causing anxiety and ultimately affecting the quality of life of PWH" reads as overly compressed and contains multiple concepts strung together without clear logical transitions. I recommend revising this statement to improve clarity and flow, for example, by separating distinct clinical and psychosocial implications into discrete, well-structured clauses.

9) We thank the reviewer for this helpful suggestion. We agree that the original sentence was overly compressed and combined multiple concepts without sufficient clarity. We have revised this statement to separate the clinical and psychosocial implications of nonsuppressible viremia into distinct, more clearly structured clauses, improving readability and flow. The revised sentence appears as follows: ***"NSV raises concerns regarding virologic failure, potential HIV transmission to partners, and ongoing immune activation due to ongoing viral gene expression. In addition, its persistence complicates ART management and can contribute to anxiety and reduced quality of life for PWH."***

10) On page 3, lines 72-74, "Its prevalence seems to be rising, likely reflecting the higher sensitivity of clinical assays and the growing population on long-term ART." When a phenomenon appears to be increasing in prevalence, it is important to consider that this trend may reflect enhanced detection rather than a true rise. As more individuals actively look for the phenomenon through increased testing, surveillance, or reporting, the likelihood of identifying and documenting cases naturally increases, potentially creating the impression of a growing prevalence even if the underlying incidence has not changed.

10) We thank the reviewer for this important clarification. We agree that an apparent increase in the prevalence of nonsuppressible viremia may reflect enhanced detection and surveillance rather than a true rise in incidence. This distinction was the intent of the original sentence, which attributed the observed increase to the higher sensitivity of contemporary clinical assays and the growing population of individuals on long-term ART.

To make this point more explicit and avoid any potential misinterpretation, we have revised the text to clearly state that the perceived rise in NSV prevalence likely reflects improved detection and ascertainment, rather than an increase in underlying incidence. Revised text: ***"Its prevalence appears to be increasing, likely reflecting improved detection due to more sensitive clinical assays and the growing population of individuals on long-term ART."***

11) On page 5, line 105, and page 26, line 544, the authors refer to the HIV-1-specific primer used for cDNA synthesis. For transparency and reproducibility, the nucleotide sequence of this primer should be provided, at minimum within the Methods section (page 26, line 544).

11) We thank the reviewer for raising this concern. The nucleotide sequences of the primers used for cDNA synthesis were made available in previous publications (PMID: 33301425 and 36602866). We added the oligo sequences in the methods section.

12) On pages 18-19 and in Figure 6, the authors assert that the NSV variant sequences remained unchanged (i.e., exhibited no measurable evolution) over the 3-year observational period; that viruses rebounding during analytical treatment interruption (ATI) were largely distinct from the NSV variants and therefore that the sources giving rise to NSV prior to ATI were unlikely to represent the source of viral rebound; and that newly seeded viral reservoirs following ATI may subsequently contribute to NSV. Although these interpretations are plausible and conceptually sound, the manner in which the data are presented does not adequately support or clearly convey these conclusions. Moreover, the analysis appears to rely on a single illustrative case, potentially rendering the findings anecdotal rather than broadly generalizable.

12) We thank the reviewer for this careful assessment and for the opportunity to clarify the evidentiary basis and scope of these conclusions.

(1) Stability of the NSV variant over time.

The conclusion that the NSV variant remained unchanged over the 3-year observational period is directly supported by the longitudinal sequence data. The dominant NSV-associated sequence detected prior to viral rebound was identical across all available time points over three years of follow-up, with no measurable sequence diversification. Moreover, as shown in Fig. 3, the defining G744A mutation severely compromises viral replicative fitness, providing a mechanistic explanation for the absence of sequence evolution over time. Together, these observations support the conclusion that the NSV variant is genetically stable during suppressive ART.

(2) Distinction between NSV variants and viruses rebounding during ATI.

The conclusion that viruses rebounding during ATI are distinct from the NSV variant is supported by both phylogenetic and functional evidence. Phylogenetic analysis demonstrates that the NSV-associated variant and rebound viruses segregate into clearly distinct branches, indicating independent genetic origins. In addition, the NSV variant is replication-defective due to its 5' Leader defect and therefore cannot plausibly give rise to replication-competent rebound virus. These data collectively support the conclusion that the sources contributing to NSV prior to ATI are unlikely to represent the source of viral rebound.

(3) Contribution of newly seeded reservoirs following ATI to subsequent NSV.

We agree with the reviewer that this statement is speculative. We have revised the text to explicitly frame this observation as a hypothesis rather than a conclusion, and to clarify that providing definitive evidence for this possibility is beyond the scope of the current study.

Finally, we acknowledge that these observations derive from a single, intensively characterized case. Such cases are rare, particularly in the context of ATI with longitudinal plasma sampling suitable for molecular analysis. The primary intent of this analysis was not to generalize the impact of viral rebound on NSV, but rather to demonstrate

the applicability of CLAWS to cure-related clinical trial settings and to illustrate how the assay can distinguish biologically distinct sources of plasma HIV-1 RNA. We have revised the manuscript to clarify this scope and to avoid overgeneralization.

13) On page 26, lines 552 and 556, the authors report using VeriFi Polymerase (PCR Biosystems) for the outer PCR and Platinum Taq High Fidelity DNA Polymerase (Thermo Fisher Scientific) for the nested PCR. The rationale for employing different polymerases at these two amplification steps should be clarified.

13) We thank the reviewer for this suggestion. The VeriFi Polymerase was selected for the outer PCR because of its higher tolerance to inhibitors, either from the plasma sample or from the cDNA synthesis product. The nested PCR can then be performed with the Platinum Taq High Fidelity DNA Polymerase since it undergoes a significant dilution of the outer PCR product, eliminating the need for more inhibitor-resistant enzymes. Of note the two polymerases have comparable fidelity. We mentioned this rationale in the methods.

Reviewer #2 (Remarks to the Author):

In a previous study (White et al., 2023), the authors identified MSD-defective proviruses (termed 5′L-defective here) as a source of non-suppressible viremia (NSV) in plasma of ART-treated participants. Although underrepresented in the overall proviral landscape, highly clonal MSD-defective viruses were shown to be the predominant drivers of NSV. The authors demonstrated that MSD-defective proviruses were not replication-competent but retained partial viral gene expression and genomic RNA packaging capacity. Importantly, defective proviruses causing NSV evaded cellular immune pressure by displaying CTL escape mutations and were not neutralized by autologous IgGs. When expressed, these defective proviruses can be recognized by the host immune system, potentially distracting CTL responses from eliminating the latent reservoir and contributing to chronic immune activation and inflammation during ART.

In the present study, Box et al. extend their previous observations to a larger cohort of participants exhibiting NSV despite effective ART. Using sequencing of the 5′L-gag and p6/protease/RT regions, the authors confirmed prior findings and added new data from pre-analytical treatment interruption (ATI), rebound, and post-ATI plasma samples. These results demonstrated that 5′L-defective viruses predominated in plasma at all time points, except during rebound when 5′L-intact viruses became predominant. To enable rapid profiling of 5′L RNA intactness, the authors developed and validated the CLAWS dPCR assay to quantify intact and defective 5′L viruses. Comparison of sequencing and dPCR results showed that CLAWS is a practical tool for clinical assessment of persistent viremia and HIV-1 cure trials.

The broad range of experimental settings used to validate the CLAWS dPCR assay is convincing and makes this paper highly relevant by presenting a new tool to explore the dynamics of residual viremia and to assess curative interventions.

We thank the reviewer for their positive comments regarding the relevance of our manuscript, and hope that the changes we have made, detailed below, address the reviewer's concerns.

Comments

1# In addition to the "claws-msd" probe and "claws-rev" primer, the CLAWS dPCR assay was designed using nearly identical primers/probes as those used in IPDA. To gain further insights into the "true" intactness of plasma viruses by assessing the presence of both 5′L and Env regions, did the authors attempt to combine CLAWS primers/probes with IPDA Env primers/probes in a multiplex format?

Although RNA processing and degradation could induce shearing that might limit the detection of full-length genomes, combining CLAWS and IPDA assays (or at least testing for correlation between 5′L and Env intactness) might have provided more informative data on plasma virus and provirus intactness. This would have

strengthened the study by clarifying whether 5′L-intact viruses in plasma also retain intact Env regions, which is critical for assessing replication competence.

1) We thank the reviewer for this thoughtful and creative suggestion. We considered combining CLAWS with IPDA envelope primers/probes to simultaneously interrogate 5' Leader and env regions. However, there are substantial technical limitations to this approach when applied to plasma HIV-1 RNA, particularly in the setting of low-level viremia. In addition to RNA degradation during isolation, reverse transcription would require efficient synthesis of long (~7 kb) cDNA molecules, and both RNA and cDNA shearing would compromise maintenance of linkage between the 5' Leader and distal env regions. These factors would markedly reduce assay sensitivity, which is critical for studying nonsuppressible viremia. Nevertheless, we tested this approach on plasma from individuals with NSV caused by intact 5'Leader RNA. The figure below shows the results from a representative participant (VC2001). When using two distant targets, while we can promptly detect env RNA, the requirement for long cDNA fragments causes a reduction in the detection of distal PSI RNA copies, preventing the detection of double positive RNA molecules.

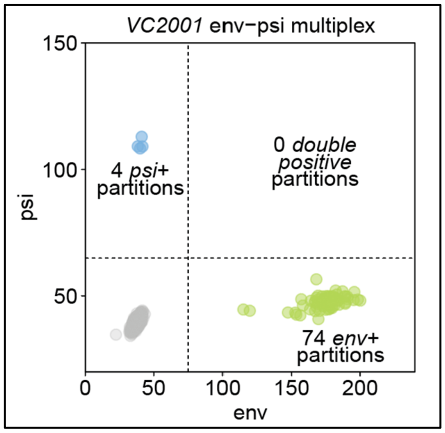

Digital PCR analysis of plasma HIV-1 RNA using the two targets from IPDA. Representative results from participant VC2001 (NSV caused by 100% MSD intact RNA). cDNA synthesis using primers in env results in robust recovery of env copies, but it results in a marked reduction of psi copies and no double positive RNA molecules, due to shearing and the lower yield of long cDNA molecules.

Additionally, we sought to answer the reviewer's question about whether the addition of a third amplicon in env would improve the discrimination of intact versus defective proviruses. We selected eight participants with additional plasma samples and performed both CLAWS and env RNA quantification in parallel by dividing the isolated RNA into two aliquots. The results, summarized in the figure to the right, show that while the concentration in plasma of total HIV-1 RNA by CLAWS (ATG) and env RNA strongly correlate, regardless of the presence of defects in the MSD, suggesting that 1) most HIV-1 RNA contributing to NSV retains the env region targeted by IPDA, and 2) adding env to the CLAWS read-out would not improve further the discrimination between intact and defective virus in plasma.

For these reasons, CLAWS was intentionally designed as a highly sensitive assay focused on the 5' Leader region, where defects directly relevant to NSV are enriched. While combined or multiplexed approaches may be feasible in samples with high HIV-1 RNA levels, they are unlikely to perform robustly in the low-copy-number plasma samples that are the focus of this study.

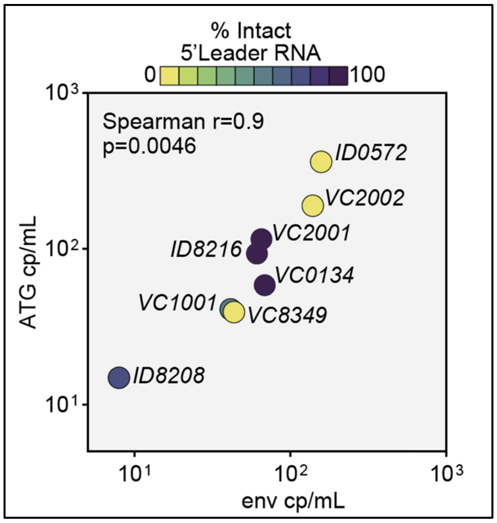

Quantification of HIV-1 in plasma by CLAWS correlates with env RNA. Circles represent plasma from each participant, and circle colors indicate the % of intact 5'L RNA by CLAWS.

2# As a corollary to Comment 1, do the authors have any evidence regarding the actual genome-wide intactness of 5′L-intact viruses detected in plasma? While the manuscript demonstrates that 5′L-defective sequences dominate NSV, it remains unclear whether the minority population of 5′L-intact sequences represents fully replication-competent virus or harbors defects in other genomic regions (e.g., env, accessory genes).

This information is critical for refining cure strategies, as it would clarify whether: 1) 5′L-intact viruses in plasma during NSV represent a persistent source of replication-competent virus. 2) These viruses could contribute to viral rebound upon ART interruption. 3) Therapeutic interventions should differentially target 5′L-intact versus 5′L-defective reservoirs

2) We thank the reviewer for this important question. We agree that genome-wide characterization of plasma HIV-1 RNA would provide the most definitive assessment of the replication competence of 5' Leader–intact variants detected during NSV. However, near–full-length sequencing of plasma-associated HIV-1 RNA is technically challenging at the low viral loads characteristic of NSV, owing to the limited efficiency of long-range PCR (PMID: 36074794), the requirement for large plasma volumes, or the need to rely on bulk amplification approaches that could obscure clonality.

Despite these limitations, several independent lines of evidence suggest that a substantial fraction of 5' Leader–intact plasma variants contributing to NSV are likely near-intact and, in some cases, replication competent. First, in our prior work (Simonetti et al., PMID: 26858442), the predominant plasma clone in an individual with NSV was shown to be infectious, as it could be recovered from quantitative viral outgrowth assays and the reconstructed proviral sequence generated infectious virus in vitro. Second, Halvas et al. (PMID: 33016926) demonstrated that in 4 of 8 individuals with NSV, plasma HIV-1 RNA sequences matched sequences recovered from viral outgrowth cultures, directly linking plasma variants to replication-competent proviruses. Third, in our recent work (Wu et al., PMID: 40802737), we identified three individuals in whom predominant plasma clones matched viral outgrowth sequences, indicating infectious proviruses as the source of NSV; these include participants ID0477 and ID0459 from the original cohort of the present study, and VC0479 from the validation cohort added in the revised manuscript.

By contrast, we have recently reported rare cases in which NSV was partially driven by proviruses harboring internal deletions outside the 5' Leader region, including defects affecting accessory genes and env. In these cases, central deletions markedly impaired viral gene expression, and productive virus expression required superinfection with intact proviruses, indicating that such genomes are unlikely to sustain NSV independently.

Taken together, these observations support a model in which NSV is primarily driven by two classes of proviruses: (i) 5' Leader–defective proviruses that retain the capacity for RNA transcription and virion production, and (ii) intact, likely infectious, proviruses with preserved 5' Leader sequences. While we cannot exclude the presence of additional, uncharacterized defects in a subset of 5' Leader–intact plasma variants, definitive resolution of this question would require full-length sequencing approaches that are beyond the scope of the present study.

3# While 5′L-defective sequences dominate HIV-1 RNA in the plasma of ART-treated participants, 5′L-intact sequences were detected in 18 of 31 participants (58%) (Figure 2A). Given that these 5′L-intact sequences, though representing a minority of total NSV, were present in the majority of participants, how can the authors rule out the possibility that this small fraction of NSV contributes meaningfully to viral persistence in ART-treated participants?

*Could these 5′L-intact viruses represent low-level replication-competent virus production?

*Do participants with detectable 5′L-intact NSV show different clinical outcomes (e.g., time to rebound after ATI, reservoir size, immune activation markers)?

*Should this population be considered clinically irrelevant to HIV-1 pathogenesis, or does it warrant closer monitoring?

3) We thank the reviewer for raising this important point. We agree that although 5' Leader–defective variants dominate plasma HIV-1 RNA during NSV, the detection of 5' Leader–intact sequences in a majority of participants indicates that this minority population may still be biologically relevant.

As discussed above, available evidence suggests that at least a subset of 5' Leader–intact plasma variants may derive from near-intact, potentially replication-competent proviruses. However, in the context of NSV, these variants typically constitute a small fraction of total plasma HIV-1 RNA and do not exhibit signatures of ongoing viral evolution, arguing against sustained rounds of replication. Rather, we interpret their presence as reflecting intermittent virus production from long-lived infected cells rather than active viral spread.

With respect to clinical outcomes, the present study was not powered to assess associations between low-level 5' Leader–intact NSV and parameters such as time to viral rebound after analytical treatment interruption, reservoir size, or immune activation. Addressing these questions will require larger, longitudinal cohorts and integration with cure-related clinical trials.

Accordingly, we do not consider 5' Leader–intact NSV to be clinically irrelevant; instead, we view it as a minority but potentially meaningful component of persistent viremia that warrants further investigation. In contrast, our data demonstrate that the bulk of NSV is driven by 5' Leader–defective variants, which fundamentally alters the interpretation of detectable plasma HIV-1 RNA in ART-treated individuals.

Finally, we revised the discussion to address the potential role of intact and 5'Leader defective proviruses to HIV-1 pathogenesis during ART.

In conclusion: This is a well-executed study that makes significant contributions to our understanding of HIV persistence and provides a valuable new tool (CLAWS) for the field. The major comments raised above, if addressed, would further strengthen the manuscript's impact and clinical utility.

We thank again the reviewer for their supportive comments.

Reviewer #3 (Remarks to the Author):

Box et al present sequence analysis of plasma HIV sequence in 32 PWH who have non-suppressible viremia (NSV), showing that a large proportion of plasma HIV RNA in this population contains 5-Leader defects that cluster around the MSD, results that expand earlier findings from the same group and others. They go on to develop a dPCR assay to quickly and easily discern MSD defects in plasma. The paper is overall well-written, the methodology sound, will be of high interest to the HIV remission field and may also have important clinical implications. Some clarification throughout the text will be helpful, particularly regarding how proportions of clonal variants relate to the overall finding that 95% of plasma viremia consists of defective variants.

We thank the reviewer for their positive comments regarding the quality and importance of our manuscript. We hope that the changes we have made, detailed below, improve the clarity of the manuscript and address the reviewer's concerns.

Major comments:

-The description in the abstract of the proportions of variants is unclear (lines 39-41). Adding the breakdown of clonal variants that contributes to the 95% of defective plasma variants may help lead into the next sentence and clarify how the variant proportions contribute to the overall amount of defectives.

1) We thank the reviewer for this helpful suggestion. We agree that the original wording did not clearly distinguish between the proportion of plasma RNA accounted for by clonal variants and the distribution of unique 5' Leader variants. We have revised the abstract to explicitly clarify how clonal defective variants contribute to the overall burden of defective plasma RNA and to improve the transition to the subsequent sentence.

Here is the new sentence: **We analyzed plasma from 32 individuals with NSV. Viremia was driven by highly clonal viral populations within each participant, with 92% of sequences being identical. In most individuals, dominant clones carrying 5′ Leader defects accounted for nearly all detectable plasma HIV-1 RNA (median, 95%). Across all participants, we recovered 91 unique 5′ Leader variants, of which 51**

carried short deletions (2–97 nucleotides) clustered around the major splice donor (MSD) or single-nucleotide mutations at conserved positions required for U1 spliceosome recognition.

Minor comments:

-In Figure 1A authors should label the red dotted line.

2) We thank the reviewer for pointing the missing label. We labeled the red dotted line, which indicates the limit of quantification of 20 HIV-1 RNA copies/mL.

-Line 143: Please define "regions of length polymorphism", or use a different term.

*3) We thank the reviewer for this suggestion. Regions of length polymorphism indicate positions with frequent nucleotide insertions or deletions (sometime referred as in/dels). To improve clarity, we revised the sentence with "**length variation**".*

-Line 186: As this is the first mention of the 91 unique variants (besides the abstract), it should be defined/included earlier in the manuscript, perhaps in the first section where variant amounts and proportions are discussed (around lines 109-110, and/or line 140 perhaps?).

4) This is a great suggestion. We added a sentence in line 113 in which we provided to total number of unique variants recovered from the 5'Lgag and p6rt data.

-Figure 3C: The labels for the White and Mohammadi papers appear to go with 3D. Consider changing the placement.

5) We thank the reviewer for this suggestion. The labels refer to both Figures 3C and 3D, which are accordingly color coded. We changed the placement of the labels to improve clarity.

-Figure 3E figure legend: Participant is labeled as 'AU0139' rather than 'ID0139'.

6) We thank the reviewer for noticing this typo. We corrected the label to 'ID0139'.

-Lines 273-274 and Fig 3: This would be better visualized if the different deletions (including delta97) were labeled on Figure 3 next to their corresponding sequences. This way reader can better visualize the deletions in relation to primer binding sites.

7) We thank the reviewer for this helpful suggestion. The deletions were already labeled adjacent to their corresponding sequences in Fig. 3C; however, to improve visibility and readability, we have revised the figure to make these labels more prominent (e.g., by bolding and increasing contrast). This change helps to visualize more clearly the location of each deletion. We also added a scheme in Figure 4B to help visualize the location of deletions relative to the probes and primers binding sites and how they affect the assay.

-Figures 4 and S7: Clarity would be enhanced by a schematic showing the genome with primer/probe binding sites for each assay.

8) We thank the reviewer for this suggestion. Kindly see the response to the next comment.

-Section starting on line 332: In addition to the comment above some additional context about what the IPDA measures vs what the CLAWS assay measures and what similarities/differences in results would be expected based on current knowledge (for example, IPDA interrogates a 3' region as well as 5', how would results be interpreted given CLAWS does not have a 3' counterpart).

9) We thank the reviewer for this helpful suggestion and agree that additional context comparing what CLAWS and IPDA measure would improve interpretability, particularly given the distinct genomic regions interrogated by the two assays.

In response, we have added a new schematic (Fig. S7) illustrating the genomic locations targeted by CLAWS and IPDA and revised the text to explicitly describe the similarities and differences between the two approaches. As now clarified, IPDA interrogates both a 5′ region and a distal 3′ region of the proviral genome to infer proviral intactness, whereas CLAWS is specifically designed to interrogate the 5′ Leader region and the beginning of the gag open reading frame.

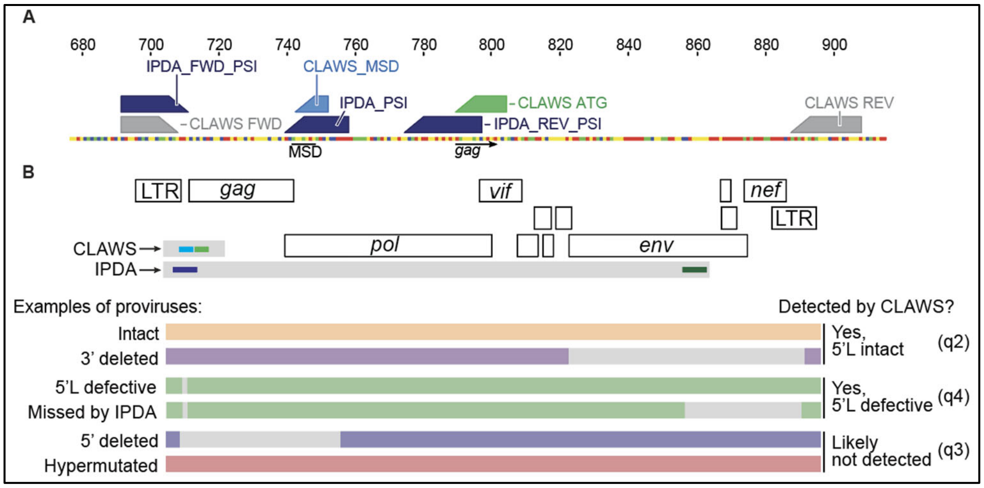

Comparison of CLAWS and IPDA. Panel A shows the location of primers and probes from both CLAWS and IPDA. Panel B shows how proviruses are scored by the two assays.

Consequently, proviruses classified as 5′ Leader–intact by CLAWS include those scored as intact by IPDA, as well as additional defective proviruses that retain an intact 5′ Leader but harbor defects elsewhere in the genome. Conversely, proviruses classified as 5′ Leader–defective by CLAWS include both genomes with isolated MSD/PSI defects and those with additional downstream defects (e.g., in env). Although CLAWS, in its current implementation, does not interrogate the distal 3′ end of the proviral genome, the frequency of 5′ Leader–defective proviruses measured by CLAWS is comparable to estimates of near-intact proviruses carrying isolated MSD/PSI defects reported in multiple near–full-length proviral sequencing studies (PMID: 27500724, 29045846, 28628034).

We revised the results section accordingly. ***"To relate these findings to the broader proviral landscape, we compared the frequency of 5'L-defective HIV-1 DNA copies measured by CLAWS with the frequency of intact and total proviruses quantified by the Intact Proviral DNA Assay[17] (IPDA). The two methods have partially overlapping primers and probes in the 5'L region (fig S7B). Additionally, proviruses are not equally scored by the two assays, given that CLAWS only targets the 5'L and the first portion of gag. For example, proviruses scored as intact or 3' deleted by IPDA would be both classified as 5'L intact by CLAWS (fig. S7C)."***

-Figure 5B: It would be helpful if the % of intact and defective 5'L sequences could be added on the left side (the sequencing results) to be more easily compared with the % that is given on the right for the CLAWS data.

10) We thank the reviewer for this suggestion. We added the percentage of 5'Leader intact RNA next to the sequencing results.

-Line 395-396: Is there data from a different participant that can be used? This participant's plasma viral RNA sequences contained a mutation that abolished detection with the CLAWS assay, necessitating participant-specific assay design. It would be nice to see data that illustrates the CLAWS assay working as designed on samples from a clinical trial. Of course, clinical samples are limited and this is not always possible. Just something to consider.

11) We thank the reviewer for this thoughtful suggestion and agree that inclusion of an ATI participant whose virus did not require probe adaptation would further strengthen the example. However, such cases are rare, given the limited number and sample size of clinical trials involving ATI and the fact that many trials restrict

enrollment to fully suppressed individuals, thereby excluding people with NSV. In this case, although a participant-specific CLAWS probe was required due to a polymorphism, we demonstrate strong concordance between this adapted CLAWS assay and single-genome sequencing, indicating that the assay performs as intended and thus serves as a proof of principle for the applicability of CLAWS in cure-related clinical studies. We agree that broader application of CLAWS in cure-related trials will be informative, and we plan to harness CLAWS to study more individuals with NSV in the context of cure-related trials, as well as post-intervention controllers showing periods of low-level fluctuating viremia, observed in many of the most recent bNAb studies (RIO, FRESH, MCA-1031, ACTG A5386, etc.).

-Line 406: There is no call-out for Fig 6F.

12) We thank the reviewer for catching the missing call-out. It has been added.

-Line 428: Do the authors mean "defective viral RNA" rather than "defective proviruses"?

13) We thank the reviewer for pointing out this ambiguity. Our intent was to emphasize that the circulating plasma virus originates predominantly from defective proviruses, but we agree that the original phrasing was confusing. We have revised the text to clearly distinguish between defective proviruses and the viral RNA transcribed from them.

Revised text: **"…RNA transcribed from defective proviruses accounted for a median of 95% of plasma HIV-1 RNA, firmly establishing their central role in persistent viremia."**

-Line 447: I suggest authors re-word the sentence starting with "Thus, these proviruses may be released from…" to enhance clarity. For example, the word "released" is a bit confusing. Perhaps something like "these proviruses may evade CPE and immune clearance, leading to positive selection over time"

14) We thank the reviewer for this suggestion. We rephrased the sentence to remove the ambiguity.

Revised text: **"Thus, these proviruses may be spared from negative selection due to cytopathic effects and immune clearance, such as antibody-dependent phagocytosis (ADPC) and cytotoxicity (ADCC), leading to positive selection over time."**

-Line 452-454: Do other proviruses retain expression of Nef? If so, 5'L defective proviruses would not necessarily have an advantage over others.

15) We thank the reviewer for this insightful question and the opportunity to clarify. We refer to a relative advantage compared with other transcriptionally active but Envelope-defective proviruses (e.g., those with large 3' deletions or hypermutation) that often lack functional Nef, whereas 5' Leader–defective proviruses can retain Nef expression and its pro-survival effects.

-Line 464-466: Authors should add "always or usually" and "could be", since there is still a possibility that detectable viral load is from replication (in cases of drug resistance). "First, periods of detectable viral load in adherent PWH should not always/usually be interpreted as evidence of replication, but rather could be virus released from defective proviruses"

16) We thank the reviewer for this comment. We fully agree with the reviewer that detectable viremia can also be caused by viral replication, and we revised the sentence as suggested.
